# Conservation of major and minor jelly-roll capsid proteins in Polinton (Maverick) transposons suggests that they are bona fide viruses

**DOI:** 10.1186/1745-6150-9-6

**Published:** 2014-04-29

**Authors:** Mart Krupovic, Dennis H Bamford, Eugene V Koonin

**Affiliations:** 1 Department of Microbiology, Institut Pasteur, Unité Biologie Moléculaire du Gène chez les Extrêmophiles, Paris 75015, France; 2 Department of Biosciences and Institute of Biotechnology, University of Helsinki, Viikki Biocenter 2, P.O. Box 56 (Viikinkaari 5)FIN-00014 Helsinki, Finland; 3 National Center for Biotechnology Information, National Library of Medicine, National Institutes of Health, Bethesda, MD 20894, USA

**Keywords:** Polintons, Mavericks, Transposable elements, Double jelly-roll fold, Capsid proteins, Virus evolution

## Abstract

**Reviewers:**

This article was reviewed by Lakshminarayan M. Iyer and I. King Jordan. For complete reviews, see the Reviewers’ Reports section.

Polintons (also known as Mavericks) and Tlr elements of *Tetrahymena thermophila* represent two families of large DNA transposons widespread in eukaryotes. Here, we show that both Polintons and Tlr elements encode two key virion proteins, the major capsid protein with the double jelly-roll fold and the minor capsid protein, known as the penton, with the single jelly-roll topology. This observation along with the previously noted conservation of the genes for viral genome packaging ATPase and adenovirus-like protease strongly suggests that Polintons and Tlr elements combine features of bona fide viruses and transposons. We propose the name ‘Polintoviruses’ to denote these putative viruses that could have played a central role in the evolution of several groups of DNA viruses of eukaryotes.

## Findings

Recently, an evolutionary connection has been established between eukaryotic transposons of the Polinton/Maverick family (hereafter Polintons) and the virophages [1,2], a group of satellite viruses that parasitize the giant viruses of the *Mimiviridae* family [3-5]. Polintons are large (15–20 kb) DNA transposons that encode a set of conserved proteins, including protein-primed type B DNA polymerase (pPolB), RVE family integrase, FtsK-like ATPase, cysteine protease, and several uncharacterized proteins [6,7]. These transposons are widely distributed in diverse unicellular and multicellular eukaryotes, attesting to their evolutionary success and/or ancient origin. We have previously pointed out that Polintons might encode a protein with the double jelly-roll fold found in the capsid proteins of viruses infecting hosts from all three domains of life [8]. Here we investigate the possible viral nature of polintons and its implications.

### The conserved polinton protein PY is a predicted major capsid protein

**Figure 1:**
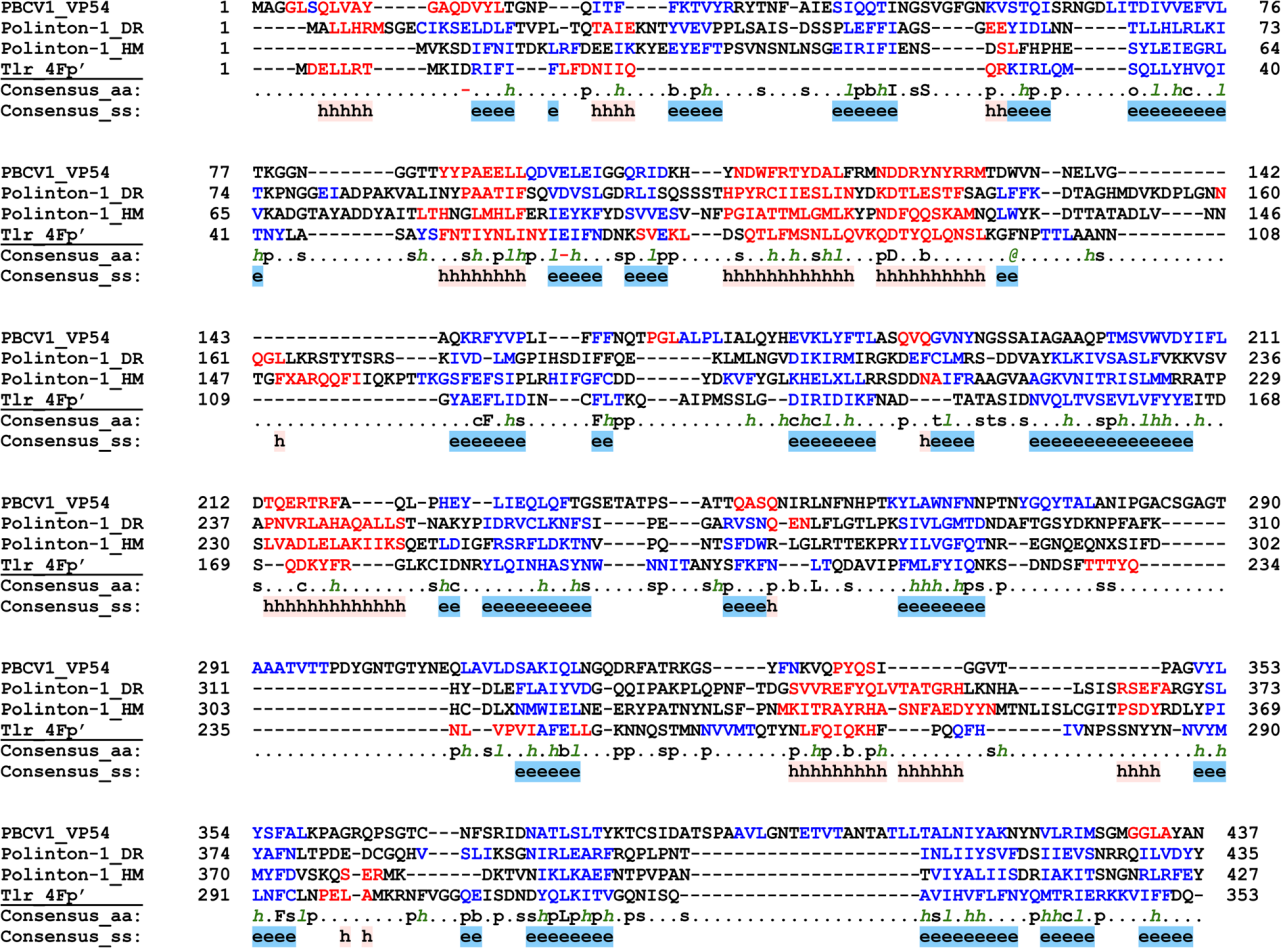
**Multiple sequence alignment of the major capsid protein VP54 of PBCV-1 with the PY proteins of Polinton 1 from*Danio rerio*(DR), Polinton 1 from*Hydra magnipapillata*(HM), and protein 4Fp’ from Tlr element.** The last two lines in each block show consensus amino acid sequence (Consensus_aa) and consensus predicted secondary structures (Consensus_ss). The protein sequences are colored according to predicted secondary structures (red: alpha-helix, blue: beta-strand). Consensus predicted secondary structure symbols: alpha-helix: h; beta-strand: e. Consensus amino acid symbols are: conserved amino acids are in uppercase letters; aliphatic (I, V, L): l; aromatic (Y, H, W, F): @; hydrophobic (W, F, Y, M, L, I, V, A, C, T, H): h; alcohol (S, T): o; polar residues (D, E, H, K, N, Q, R, S, T): p; tiny (A, G, C, S): t; small (A, G, C, S, V, N, D, T, P): s; bulky residues (E, F, I, K, L, M, Q, R, W, Y): b; positively charged (K, R, H): +; negatively charged (D, E): −; charged (D, E, K, R, H): c.

We collected a non-redundant dataset of 72 Polintons (see Methods and Additional file 1: Table S1) and systematically analyzed their proteins using HHpred [9]. This analysis showed that a conserved Polinton protein, previously denoted PY [6], is homologous to the major capsid protein (MCP) VP54 of *Paramecium bursaria Chlorella* virus 1 (PBCV-1), despite less than 15% pairwise sequence identity. The PY proteins from different Polintons produced somewhat variable repertoires of significant hits in HHpred. For example, PY from the Polinton of *Hydra magnipapillata* retrieved VP54 of PBCV-1 as the only significant hit with the probability of 97.5. By contrast, PY from *Danio rerio* Polinton (P-1_DR), in addition to VP54 (P = 97.8), also recovered the hexon protein of adenovirus, albeit with lower probability (P = 92.1). VP54 has a typical double jelly-roll (DJR) topology [10] and is conserved across *Megavirales*[11,12]; however, structurally related MCPs are also found in many smaller dsDNA viruses [13], including virophages [14]. Multiple sequence alignment of the PY and VP54 proteins revealed the conservation of all secondary structure elements in the Polinton proteins when compared to VP54 (Figure 1).

**Figure 2:**
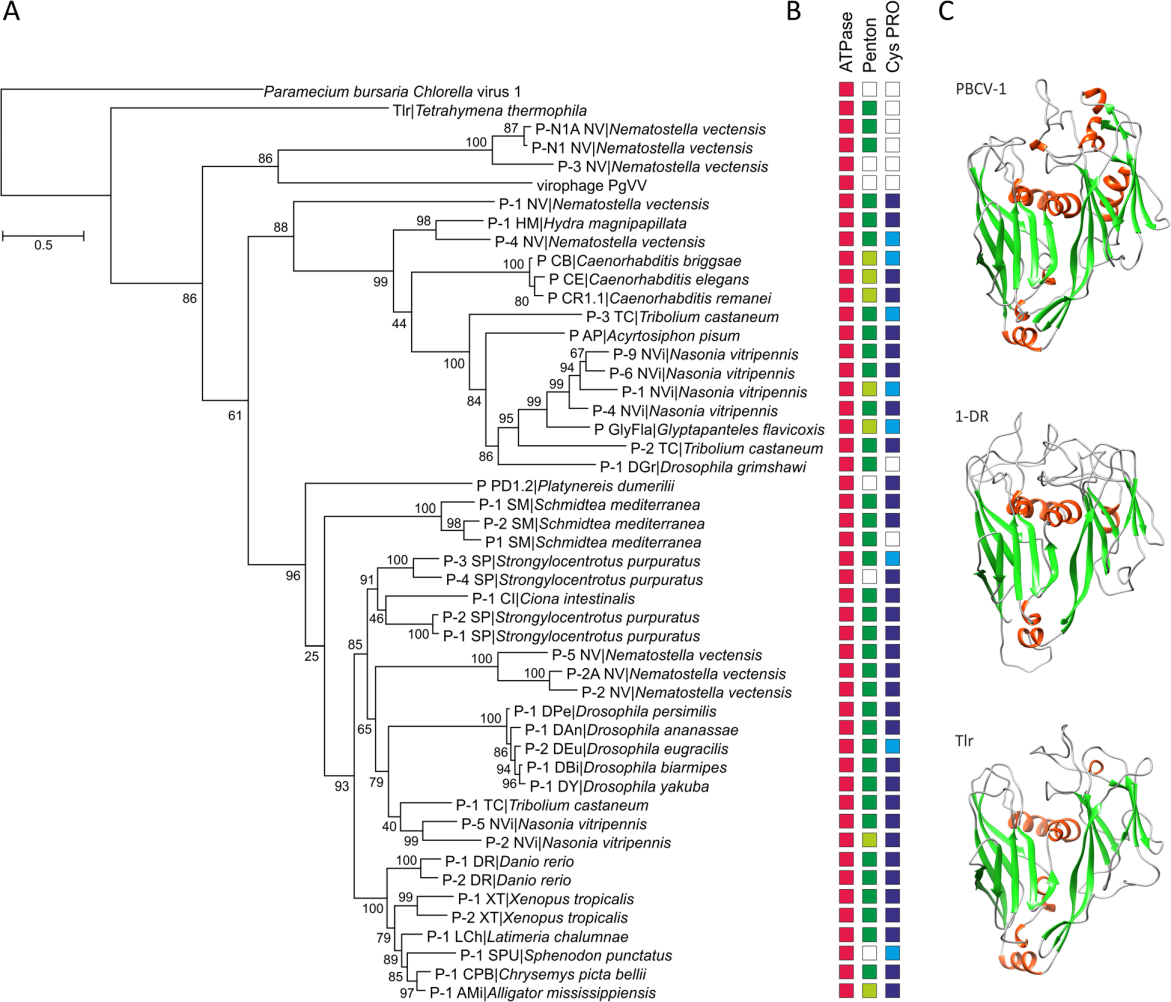
**Putative major capsid proteins of Polintons, Tlr elements and the PgVV virophage. A**. Maximum likelihood phylogenetic analysis of the putative DJR MCPs. Numbers at the branch points represent SH-like local support values. **B**. Conservation of the genes for the genome packaging ATPase (red boxes), penton protein (green boxes), and adenoviral protease (blue boxes) in Polintons containing intact MCP genes. The presence of the corresponding split genes is indicated with lighter shading, and absence of identifiable genes is shown with empty boxes. The full information on the gene presence-absence pattern is provided in Additional file 1: Table S1. **C**. Structural models of the Polinton and Tlr MCP genes along with the X-ray structure of the VP54 of PBCV-1 (PDB ID: 1M3Y). The models are colored according to the secondary structure elements: green, beta-strands; red, alpha-helixes).

To further examine the relationship between the PY proteins and viral capsid protein known to adopt the DJR fold, we used structural modeling followed by the quality assessment of the resultant model. The PY protein of P-1_DR was chosen as the target, and the X-ray structure of VP54 (PDB ID: 1M3Y) was used as the template. The quality of the generated model was found to be as good as that of the template structure (Additional file 2: Figure S1), indicating that the structure of the PY protein of P-1_DR is compatible with the DJR topology (Figure 2). This protein contains all structural elements of the DJR (Figure 1) and shows no apparent structural distortion, indicating that PY is the functional MCP of the polintons.

We next assessed the conservation of the Polinton MCP-coding genes and found that 46 of the 72 Polintons in the analyzed representative set encoded full-length MCPs (Additional file 1: Table S1), whereas in 13 other polintons the MCP-coding genes were split. Thus, more than 80% of the Polintons contain recognizable intact or fragmented MCP genes. This high conservation of the MCP proteins mirrors the conservation of the FtsK-like ATPase and cysteine protease (PRO), the two other viral-like proteins encoded by Polintons. The Polinton ATPase belongs to the family of genome-packaging ATPases of viruses with DJR MCPs [15,16], whereas PRO is homologous to the virion maturation protease encoded by adenoviruses, virophages and many Nulceocytoplasmic Large DNA Viruses (NCLDV) of eukaryotes [2]. The ATPase and PRO genes were identifiable in 83% and 74% of Polintons, respectively (Additional file 1: Table S1). Nine of the elements that did not encode MCPs also lacked the genes for both PRO and ATPase (Additional file 1: Table S1), suggestive of a concerted loss of genes implicated in the putative capsid morphogenesis. It therefore appears that among Polintons there is a continuity between viral and transposon states reminiscent of the well-studied Mu-like bacteriophages as well as metaviruses (also known as *Ty3-gypsy* retrotransposons) and pseudoviruses (also known as *Ty1-copia* retrotransposons) of eukaryotes that also combine the properties of both types of selfish elements [17-19].

### Conserved polinton protein PX is a homolog of viral penton proteins

Viruses with the DJR MCPs use a common blueprint for capsid construction: pseudohexagonal trimers of the MCP are arrayed as triangular plates to form the icosahedral facets, whereas the five-fold vertices of the capsid are occupied by a different type of capsomer [8,20-22]. In all the cases when structural information is available, the latter capsomers, called pentons in adenoviruses, display a single jelly-roll fold [14,20,23-25] and are typically encoded in the proximity of the MCP. For example, in all virophages penton protein homologs (minor capsid proteins) are encoded immediately upstream of the MCP [2]. To investigate whether a corresponding protein is also conserved in Polintons, the penton protein sequence of Organic Lake Virophage (OLV8; [26]) was used as a seed in the Blastp search against the database of Polinton proteins. As a result, a match to the conserved Polinton protein PX [6] from *Xenopus tropicalis* was obtained (24% identity over 119 aa region; E = 3e-02). We further assessed the relationship between the penton proteins of virophages and PX proteins of Polintons by constructing multiple sequence alignment and inspecting the correspondence between the secondary structure elements among the aligned sequences (Additional file 3: Figure S2). Despite the overall low sequence similarity, which appears to be a general trend even among more closely related viruses [2], the correspondence of the secondary structure between the virophage and Polinton proteins is obvious. Further evidence indicating that PX represents the penton protein of Polintons comes from the conserved genomic location of the PX-coding gene which in Polintons is typically found either immediately upstream (e.g., P-9_NVi) or more commonly one gene away from the MCP gene. Taken together, the conserved sequences and secondary structures as well as positional gene conservation lead us to conclude that Polinton protein PX is a genuine penton protein.

### Tlr elements also encode both the major and minor capsid proteins

Tlr elements found in the germline genome of the ciliate *Tetrahymena thermophila* represent a distinct type of large eukaryotic DNA transposons [27]. They share with Polintons the genes for RVE family integrase and FtsK-like ATPase but instead of pPolB, encode a superfamily 1 helicase, which displays phylogenetic affinity to the homologous proteins from certain *Megavirales* as well as transpovirons [2,28]. The presence of several viral-like genes, most notably the putative genome packaging ATPase, in the Tlr prompted us to investigate this element for the presence of the potential capsid protein gene(s). Indeed, we found that a protein (4Fp’) encoded by an open reading frame starting 78 codons upstream of the originally predicted gene 4F and completely encompassing it in frame, encodes a putative MCP of Tlr. When the 4Fp’ sequence was used as a query for the HHpred search, PBCV-1 VP54 was retrieved as the only significant hit with a probability P = 98.1. Alignment of the Tlr 4Fp’ sequence with those of the Polinton and PBCV-1 MCPs showed that the proteins align throughout their length and the secondary structure elements critical for the DJR fold are conserved (Figure 1). As in the case of P_1-DR MCP, the structural model of the 4Fp’ protein (Figure 2) displayed good stereochemical quality (Additional file 2: Figure S1), confirming that it is the DJR MCP of Tlr.

Furthermore, a Tblastn search against the Tlr sequence seeded with the putative Polinton penton proteins detected moderately significant similarity (E = 3e-04) between the predicted P-9_NVi penton protein and the product of a previously unannotated ORF of 227 codons located between the Tlr genes 7R and 1F [27]. Reciprocally, the putative penton protein of Tlr retrieved the corresponding protein of P-9_NVi as the best hit from the database of all Polinton proteins. Taken together, these results strongly suggest that, similar to the Polintons, the transposable element Tlr encodes not only the putative viral genome-packaging ATPase but also the MCP and penton proteins.

### A homologue of the Polinton MCP is encoded by the *Phaeocystis globosa* virus PgV-16T-associated virophage

Recently, a virophage-like element, PgVV, associated with the virus PgV-16T infecting *Phaeocystis globosa* has been described [29]. PgVV has been reported to lack a capsid protein gene and to replicate as a linear plasmid [29]. Given that many MCPs are highly divergent, we analyzed the PgVV for the presence of putative capsid protein genes by performing blastp searches with the Polinton MCPs as seeds. The best hit was obtained between the MCP of Polinton 3 (P-3_NV) from *Nematostella vectensis* and the PgVV protein PgVV_00012 (YP_008059897, 25% identity over 194 aa region, E = 0.01). This relationship was further validated by the evaluation of the multiple sequence alignment of PgVV_00012 with the MCPs of P-3_NV and PBCV-1 and subsequent assessment of the correspondence between their secondary structure elements. Like in the case of MCPs of polintons, the predicted secondary structure of PgVV_00012 was found to be consistent with the DJR topology (Additional file 4: Figure S3).

To better understand the relationship between the MCPs of Polintons, the Tlr element and the PgVV virophage, a maximum likelihood tree was constructed and rooted with the PBCV-1 MCP (Figure 2). According to this analysis, the MCP of the Tlr element forms an outgroup to the Polinton MCPs, suggesting that although Tlr and Polintons probably share a common origin, their divergence is an ancient event, in contrast to the previous suggestion that Tlr is a non-autonomous derivative of the Polintons [6]. The MCP of PgVV formed a clade with the Polintons from *N. vectensis* (Figure 2), in agreement with the previous analysis that presented several lines of evidence of virophage evolution from Polintons [2]. This conclusion contrasts the original proposal that virophages were ancestors of polintons that in large part rested on the assumption that virophages unlike Polintons are bona fide viruses [1]. The demonstration that most of the Polintons encompass genes for two capsid proteins invalidates this argument.

## Conclusions

Capsid proteins are among the viral hallmark proteins [30], and their presence distinguishes viruses from other types of mobile genetic elements [19,31,32]. Here we show that Polintons and Tlr elements, currently classified as non-viral transposable elements, encode two key proteins required for virion formation, the DJR MCP and the penton protein, i.e. the major and minor capsid proteins. This finding combined with previous observations that these elements also encode a typical viral genome-packaging ATPase and adenovirus-like protease (absent in Tlr) make a strong case that Polintons and Tlr elements comprise a group of genuine viruses that we propose to denote ‘Polintoviruses’. Polintoviruses might have played key roles in the evolution of DNA viruses of eukaryotes, in particular adenoviruses, virophages, and possibly the NCLDV. Identification of actively reproducing Polintoviruses is an important experimental challenge.

## Methods

The Polinton nucleotide sequences were acquired from the Repbase Update database [33], which provides the consensus sequences of closely related transposons, thereby precluding redundancy. The dataset was further complemented with previously reported Polinton sequences [7,34]. Polinton-like elements shorter than 10 kb were considered incomplete and removed, resulting in the final dataset of 72 non-redundant Polinton sequences. Distant homology detection was performed using HHpred [9]. Fasta-formatted sequences of the major and minor capsid proteins of polintoviruses discussed in this study can be found in Additional file 5. Structural modelling was performed using Modeller v9.7 [35], essentially as described previously [36]. The model was then verified for stereochemical consistency using ProSA-web [37]. Protein sequences were aligned using Promals3D [38]. For phylogenetic analysis, gapped columns (more than 30% of gaps) and columns with low information content were removed from the alignment [39]. Maximum likelihood analysis was carried out by using PhyML 3.1 [40], with the WAG model of amino acid substitution, including a gamma law with 4 substitution rate categories.

## Reviewers’ reports

### Reviewer 1: Lakshminarayan M. Iyer (National Center for Biotechnology Information, National Library of Medicine, national Institute of Health)

The detection of the jelly roll capsids in various transposons is an important discovery and it clarifies the origins of the FtsK/HerA family ATPase-containing transposons that were previously suspected to be derivatives of virophages. They are now shown to be bonafide viruses. Understanding the biology of these elements is an exciting prospect for experimental studies. I have reproduced the results independently and confirm the sequence relationships mentioned in the text. Below are a few comments that the authors might find useful.

1. In general, it would be helpful if genbank ids or accession numbers of proteins described in the text are provided either in brackets next to the protein name (as was done in one instance) or in the supplementary table. The same applies to the alignment and tree figures in the main text and supplement, where they could either expand the species names or provide gis/accession numbers in the figure.

*Authors’ response: In many cases, the major and minor capsid proteins were not properly annotated. Therefore, gis/accession numbers cannot be added. In the revised version of the manuscript we have included a new Additional file*5*which contains fasta-formatted sequences of all major and minor capsid proteins of polintoviruses discussed in this study. Furthermore, as suggested by the reviewer, we have expanded the species names in Figure*2*. In the other figures, the species names are indicated in the figure legends.*

2. On page 3, the authors write “To further address the possibility that the PY proteins adopt the DJR fold, we used structural modeling followed by the assessment of the resultant model.” Structural modeling as done in this study is not really a reliable assessment tool for homology. Unrelated sequences have been wrongly fitted on to structures in other studies. The evidence provided in the previous paragraph is sufficient to establish a sequence relationship.

Authors’ response: This is a debatable issue. Certainly, unrelated sequences have been claimed to fit a particular structure on multiple occasions (just as unrelated sequences have been aligned to falsely claim homology). Nevertheless, we tend to believe that the quality of the model matters. This brief article, in any case, is not the place to plunge into such a general methodological debate, so we changed the language in question to make the text more neutral, in particular “To further examine the relationship between the PY proteins and viral capsid protein known to adopt the DJR fold, we used structural modeling…”

3. The relationship of PgVV_00012 to the capsid jelly rolls is not written in a convincing way. The authors should provide appropriate statistics.

*Authors’ response: In the revised manuscript we have provided the E value for the hit between PgVV_00012 and the PY protein of P-3_NV (which is 0.01). We also pointed out that the predicted secondary structure of PgVV_00012 is consistent with the DJR topology, as is evident from the alignment shown in Additional file*4*: Figure S3.*

4. The sequence of the Tlr penton protein is not provided. The supplementary alignment (S2) only shows one Tlr protein (which one is it?). The sequence might either be shown in an alignment or separately in the supplement.

*Authors’ response: All protein sequences shown in the former Figure S2 (currently Additional file*3*: Figure S2) correspond to the penton proteins. Fasta-formatted sequences of all polinton proteins (including the one from Tlr) shown in the figure are now provided in the Additional file*5*.*

5. It would help a reader if the major clades were labeled in Figure 2. This can be done either by using brackets or colored branches.

Authors’ response: Although we appreciate the suggestion, we think that further modifications to the figure would make it excessively crowded, especially now that the species names were expanded.

### Reviewer 2: I.King Jordan (Department of Biology, Georgia Institute of Technology)

In this Discovery Note, Krupovic et al. make the case that two families of repetitive sequence elements, the Polintrons (Mavericks) and Tlr elements, formerly thought to be DNA transposons are more likely to be bona fide DNA viruses. The evidence in support of this assertion consists of deep sequence conservation and structural comparisons revealing that these families of genomic elements encode both major and minor viral capsid proteins, with the major capsid protein adopting the canonical jelly-roll structural topology. Such capsid proteins have previously been designated as features that distinguish viruses from transposons. This work reported here appears to be technically sound and the reasoning behind the authors’ argument for considering these mobile genetic element families is compelling.

My only suggestion is that they consider adding a figure to the paper that is a scheme illustrating the relationships between what they are designating as ‘Polintoviruses’ here and other families of DNA viruses in eukaryotes. Such an illustration could help to clarify the argument they make based on the tree topology shown in Figure 2 and may also underscore the continuity between viral and transposon states that they allude to in the manuscript.

Authors’ response: *We certainly appreciate the importance of the relationships between Polintoviruses and other viral families but have to note that this is a major subject that goes far beyond the scope of the present brief note. We are preparing a new manuscript that will be dedicated to the implications of these relationships for the evolution of viruses.*

## Competing interests

The authors declare that they have no competing interests.

## Authors’ contributions

MK collected the data; MK and EVK analyzed the data; MK, DHB and EVK wrote the manuscript. All authors read and approved the final version.

## Supplementary Material

Additional file 1: Table S1 Properties of the analyzed Polintons. Click here for file

Additional file 2: Figure S1 Quality assessment of the three-dimensional models. Quality of the generated models along with that of the template structure was evaluated using PsoSA-web at https://prosa.services.came.sbg.ac.at/prosa.php. The calculated quality (Z) scores (closed circles) are displayed in the context of the Z-scores of all experimentally determined protein structures available in the Protein Data Bank. Every dot represents a distinct structure solved by X-ray crystallography (light blue) or NMR (dark blue). PBCV-1, *Paramecium bursaria Chlorella* virus 1 (Z-score: −6.09); P1-DR, Polinton 1 from Danio rerio (Z-score: −6.84); Tlr, Tlr element from *Tetrahymena thermophila* (Z-score: −6.05). Click here for file

Additional file 3: Figure S2 Multiple sequence alignment of virophage penton base proteins with the PX proteins from Polinton and Tlr elements. The last two lines in each block show consensus amino acid sequence (Consensus_aa) and consensus predicted secondary structures (Consensus_ss). Representative sequences have magenta names and they are colored according to predicted secondary structures (red: alpha-helix, blue: beta-strand). If the sequences are in aligned order, the sequences with black names directly under a representative sequence are in the same pre-aligned group. Consensus predicted secondary structure symbols: alpha-helix: h; beta-strand: e. Consensus amino acid symbols are: conserved amino acids are in uppercase letters; aliphatic (I, V, L): l; aromatic (Y, H, W, F): @; hydrophobic (W, F, Y, M, L, I, V, A, C, T, H): h; alcohol (S, T): o; polar residues (D, E, H, K, N, Q, R, S, T): p; tiny (A, G, C, S): t; small (A, G, C, S, V, N, D, T, P): s; bulky residues (E, F, I, K, L, M, Q, R, W, Y): b; positively charged (K, R, H): +; negatively charged (D, E): −; charged (D, E, K, R, H): c. The alignment was constructed with PROMALS3D (http://prodata.swmed.edu/promals3d). Abbreviations: AP, *Acyrtosiphon pisum*; NV, *Nematostella vectensis*; NVi, *Nasonia vitripennis*; TC, *Tribolium castaneum*; CI, *Ciona intestinalis*; SP, *Strongylocentrotus purpuratus*; XT, *Xenopus tropicalis*; CPB, *Chrysemys picta bellii*. Click here for file

Additional file 4: Figure S3 Multiple sequence alignment of the major capsid proteins of PBCV-1 and Polinton 3 of Nematostella vectensis (P3_NV) with the protein 00012 of the virophage PgVV. The last two lines in each block show consensus amino acid sequence (Consensus_aa) and consensus predicted secondary structures (Consensus_ss). The protein sequences are colored according to predicted secondary structures (red: alpha-helix, blue: beta-strand). Consensus predicted secondary structure symbols: alpha-helix: h; beta-strand: e. Consensus amino acid symbols are: conserved amino acids are in uppercase letters; aliphatic (I, V, L): l; aromatic (Y, H, W, F): @; hydrophobic (W, F, Y, M, L, I, V, A, C, T, H): h; alcohol (S, T): o; polar residues (D, E, H, K, N, Q, R, S, T): p; tiny (A, G, C, S): t; small (A, G, C, S, V, N, D, T, P): s; bulky residues (E, F, I, K, L, M, Q, R, W, Y): b; positively charged (K, R, H): +; negatively charged (D, E): −; charged (D, E, K, R, H): c. The alignment was constructed with PROMALS3D (http://prodata.swmed.edu/promals3d). Click here for file

Additional file 5 Fasta-formatted sequences of the predicted major and minor capsid proteins of polintoviruses discussed in this study. Click here for file
