# Supplementary material for: Conservation of major and minor jelly-roll capsid proteins in Polinton (Maverick) transposons suggests that they are bona fide viruses
Source: Biol Direct. 2014 Apr 29;9:6. doi: 10.1186/1745-6150-9-6 (PMC4028283; doi:10.1186/1745-6150-9-6)
Supplement: Additional file 1: Table S1 — Properties of the analyzed Polintons. [file 1745-6150-9-6-S1.pdf]

Table S1. Properties of the analyzed Polintons.

Polinton name	Host	Size, bp	MCP (PY)	Penton (PX)	ATPase	Protease
Polinton_AP	*Acyrtosiphon pisum*	33814	+	+	+	+
Polinton-1_AMi	*Alligator mississippiensis*	14974	+	split	+	+
Polinton-2_AMi	*Alligator mississippiensis*	15555	fragmented	split	+	+
P_Cb	*Caenorhabditis briggsae*	15423	+	fragmented	+	truncated
Polinton-1_CB	*Caenorhabditis briggsae*	16633	fragmented	fragemented	+	+
Polinton-2_CB	*Caenorhabditis briggsae*	15471	fragmented	fragmented	+	+
P_Ce	*Caenorhabditis elegans*	17274	+	fragmented	+	+
P_Cr1.1	*Caenorhabditis remanei*	15239	+	fragmented	+	+
Polinton-1_CPB	*Chrysemys picta bellii*	13369	+	+	+	+
Polinton-1_CI	*Ciona intestinalis*	15061	+	+	+	+
Polinton-2_CI	*Ciona intestinalis*	13695	fragmented	+	+	+
P_CotSesBraco	*Cotesia sesamiae Mombasa bracovirus*	48885	fragmented	-	-	truncated
Polinton-1_CGi	*Crassostrea gigas*	20773	fragmented	truncated	+	+
P_Dr	*Danio rerio*	15460	-	-	-	-
Polinton-1_DR	*Danio rerio*	18485	+	+	+	+
Polinton-1N1_DR	*Danio rerio*	15469	-	-	-	-
Polinton-2_DR	*Danio rerio*	16276	+	+	+	+
Polinton-2N1_DR	*Danio rerio*	11092	-	-	-	-
Polinton-1_DAn	*Drosophila ananassae*	17900	+	+	+	+
Polinton-1_DBi	*Drosophila biarmipes*	13688	+	+	+	+
Polinton-1_DEl	*Drosophila elegans*	10296	fragmented	+	inactivated	+
Polinton-1_DEu	*Drosophila eugracilis*	10967	fragmented	+	+	+
Polinton-2_DEu	*Drosophila eugracilis*	11524	+	+	+	+
Polinton-1_DGr	*Drosophila grimshawi*	15303	+	+	+	-
Polinton-1_DPe	*Drosophila persimilis*	15797	+	+	+	+
Polinton-1_DY	*Drosophila yakuba*	14782	+	+	+	+
Polinton-1_EI	*Entamoeba invadens*	16504	-	-	-	-
P_Gi1.1	*Glomus intraradices*	11954	-	-	-	-
P_Gi1.2	*Glomus intraradices*	11019	-	-	+	-
P_GlyFla	*Glyptapanteles_flavicoxis*	26448	+	split	+	truncated
Polinton-1_HM	*Hydra magnipapillata*	20689	+	+	+	+
Polinton-2_HM	*Hydra magnipapillata*	42550	-	-	-	-
Polinton-3_HM	*Hydra magnipapillata*	38791	-	-	-	-
Polinton-1_LCh	*Latimeria chalumnae*	17525	+	+	+	+
Polinton-1_NVi	*Nasonia vitripennis*	14499	+	truncated	+	truncated
Polinton-3_NVi	*Nasonia vitripennis*	12588	fragmented	+	+	truncated
Polinton-4_NVi	*Nasonia vitripennis*	12608	+	+	+	+
Polinton-5_NVi	*Nasonia vitripennis*	18379	+	+	+	+
Polinton-6_NVi	*Nasonia vitripennis*	10272	+	+	+	+
Polinton-8_NVi	*Nasonia vitripennis*	16996	fragmented	+	+	+
Polinton-9_NVi	*Nasonia vitripennis*	17121	+	+	+	+
Polinton-1_NV	*Nematostella vectensis*	17653	+	+	+	+
Polinton-2_NV	*Nematostella vectensis*	21301	+	+	+	+
Polinton-2A_NV	*Nematostella vectensis*	20836	+	+	+	+
Polinton-3_NV	*Nematostella vectensis*	16575	+	-	+	-

Polinton-4_NV	*Nematostella vectensis*	13070	+	+	+	truncated
Polinton-5_NV	*Nematostella vectensis*	19102	+	+	+	+
Polinton-N1_NV	*Nematostella vectensis*	14761	+	+	+	-
Polinton-N1A_NV	*Nematostella vectensis*	14355	+	+	+	-
Polinton-N1B_NV	*Nematostella vectensis*	15049	fragmented	+	+	-
P_Od1.1	*Oikopleura dioica*	22700	fragmented	-	-	+
P_Od1.2	*Oikopleura dioica*	24732	+	-	+	-
Polinton-1_PI	*Phytophthora infestans*	18398	-	+	+	inactivated
P_Pd1.1	*Platynereis dumerilii*	10777	-	-	+	-
P_Pd1.2	*Platynereis dumerilii*	27467	+	-	+	+
Polinton-1_GI	*Rhizophagus intraradices*	11954	-	-	-	-
Polinton1_SM	*Schmidtea mediterranea*	12786	+	+	+	-
Polinton-1_SM	*Schmidtea mediterranea*	14867	+	+	+	+
Polinton2_SM	*Schmidtea mediterranea*	11578		+	+	-
Polinton-2_SM	*Schmidtea mediterranea*	15944	+	+	+	+
Polinton-1_SPU	*Sphenodon punctatus*	11940	+	-	+	inactivated
Polinton-1_SP	*Strongylocentrotus purpuratus*	16918	+	+	+	+
Polinton-2_SP	*Strongylocentrotus purpuratus*	14353	+	+	+	+
Polinton-3_SP	*Strongylocentrotus purpuratus*	16510	+	+	+	truncated
Polinton-4_SP	*Strongylocentrotus purpuratus*	15575	+	-	+	+
Polinton-5_SP	*Strongylocentrotus purpuratus*	16525	fragmented	+	+	+
Polinton-1_TC	*Tribolium castaneum*	13486	+	+	+	+
Polinton-2_TC	*Tribolium castaneum*	16981	+	+	+	+
Polinton-3_TC	*Tribolium castaneum*	17681	+	+	+	truncated
Polinton-1_TV	*Trichomonas vaginalis*	20724	-	-	-	-
Polinton-1_XT	*Xenopus tropicalis*	13692	+	+	+	+
Polinton-2_XT	*Xenopus tropicalis*	14828	+	+	+	+
