# Supplementary material for: Conservation of major and minor jelly-roll capsid proteins in Polinton (Maverick) transposons suggests that they are bona fide viruses
Source: Biol Direct. 2014 Apr 29;9:6. doi: 10.1186/1745-6150-9-6 (PMC4028283; doi:10.1186/1745-6150-9-6)
Supplement: Additional file 2: Figure S1 — Quality assessment of the three-dimensional models. Quality of the generated models along with that of the template structure was evaluated using PsoSA-web at https://prosa.services.came.sbg.ac.at/prosa.php. The calculated quality (Z) scores (closed circles) are displayed in the context of the Z-scores of all experimentally determined protein structures available in the Protein Data Bank. Every dot represents a distinct structure solved by X-ray crystallography (light blue) or NMR (dark blue). PBCV-1, Paramecium bursaria Chlorella virus 1 (Z-score: −6.09); P1-DR, Polinton 1 from Danio rerio (Z-score: −6.84); Tlr, Tlr element from Tetrahymena thermophila (Z-score: −6.05). [file 1745-6150-9-6-S2.pdf]

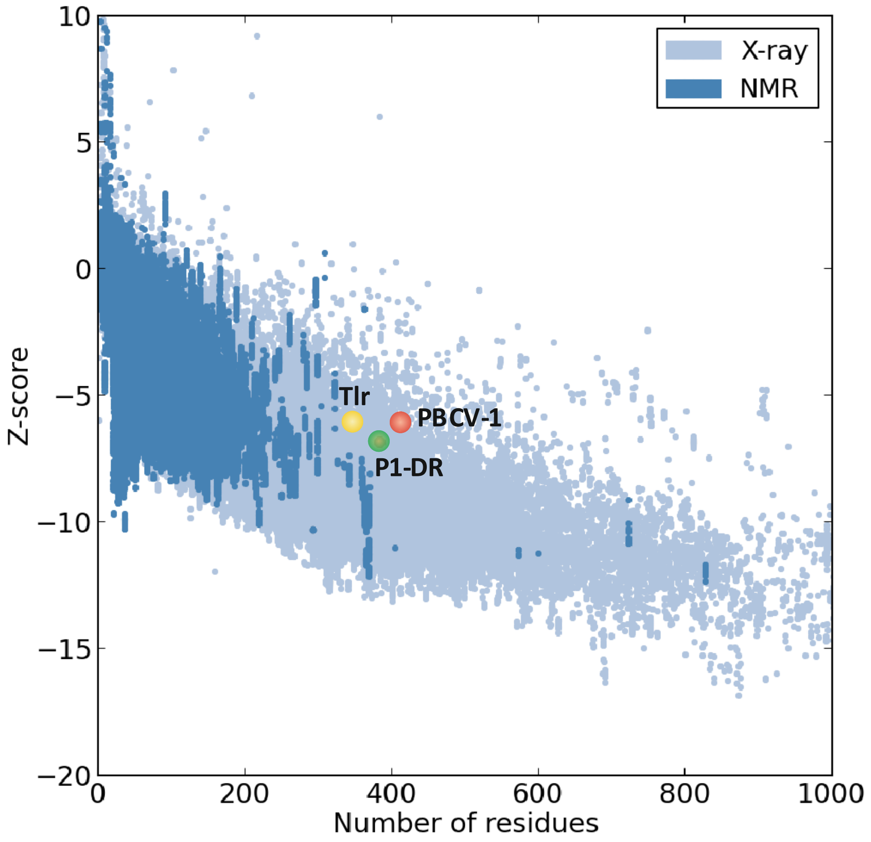

Figure S1. Quality assessment of the three-dimensional models. Quality of the generated models along with that of the template structure was evaluated using PsoSA-web at https://prosa.services.came.sbg.ac.at/prosa.php. The calculated quality (Z) scores (closed circles) are displayed in the context of the Z-scores of all experimentally determined protein structures available in the Protein Data Bank. Every dot represents a distinct structure solved by X-ray crystallography (light blue) or NMR (dark blue). PBCV-1, *Paramecium bursaria Chlorella* virus 1 (Z-score: -6.09); P1-DR, Polinton 1 from *Danio rerio* (Z-score: -6.84); Tlr, Tlr element from *Tetrahymena thermophila* (Z-score: -6.05).
