# Supplementary material for: Conservation of major and minor jelly-roll capsid proteins in Polinton (Maverick) transposons suggests that they are bona fide viruses
Source: Biol Direct. 2014 Apr 29;9:6. doi: 10.1186/1745-6150-9-6 (PMC4028283; doi:10.1186/1745-6150-9-6)
Supplement: Additional file 3: Figure S2 — Multiple sequence alignment of virophage penton base proteins with the PX proteins from Polinton and Tlr elements. The last two lines in each block show consensus amino acid sequence (Consensus_aa) and consensus predicted secondary structures (Consensus_ss). Representative sequences have magenta names and they are colored according to predicted secondary structures (red: alpha-helix, blue: beta-strand). If the sequences are in aligned order, the sequences with black names directly under a representative sequence are in the same pre-aligned group. Consensus predicted secondary structure symbols: alpha-helix: h; beta-strand: e. Consensus amino acid symbols are: conserved amino acids are in uppercase letters; aliphatic (I, V, L): l; aromatic (Y, H, W, F): @; hydrophobic (W, F, Y, M, L, I, V, A, C, T, H): h; alcohol (S, T): o; polar residues (D, E, H, K, N, Q, R, S, T): p; tiny (A, G, C, S): t; small (A, G, C, S, V, N, D, T, P): s; bulky residues (E, F, I, K, L, M, Q, R, W, Y): b; positively charged (K, R, H): +; negatively charged (D, E): −; charged (D, E, K, R, H): c. The alignment was constructed with PROMALS3D (http://prodata.swmed.edu/promals3d). Abbreviations: AP, Acyrtosiphon pisum; NV, Nematostella vectensis; NVi, Nasonia vitripennis; TC, Tribolium castaneum; CI, Ciona intestinalis; SP, Strongylocentrotus purpuratus; XT, Xenopus tropicalis; CPB, Chrysemys picta bellii. [file 1745-6150-9-6-S3.pdf]

```
Sputnik_V18-V19     1  MSYSHSIKDCQEPDTVYYDILIP--FKPNDQ--GFS----PAIFQAQLTQPIVHN-PSEYFLSVVRFSIPTQNIPLTIPQIQPYPNT   78
Mavirus_MV17        1  MK--------------QY-IWLNETIKSNKQLAGPRGSYKRPVSVDIFRSSTILDPDKNYLLIVEEFHLHKIRL-----------PL   61
Tlr_penton          1  MN---------------Y-IFID-SSKINP---SCF-----YNFQVQLNQKVRVT----KYIKLIKAVIPYDDY-----------LI   47
P_AP_PX             1  MH--------------ES-ITLS--FTGNS-----------TVLSANYFPSLNVY--GDSEIALLSLQTFNSFP----------NII   47
P-1_NV_PX           1  MEN----------IVERA-VYIS--SRDREARGVSRP----DDFTIKMNPSLRLSNDARHEIAVDKISMTYSWH----------NIN   60
P-4_NV_PX           1  MEQLLE--KIARNTEPKKSFYIL--VSDK-----------STRIRTKFNPLLQLDKSKQYEMALVNLETYYSFP-----------NI   61
P-4_NVi_PX          1  ME--------------DS-LTLS--LSGT-----------SAVLEAQYFPPLELSANKSYVLGLVELLTFNSIP-----------NI   48
P-3_NVi_PX          1  MI--------------ES-LTLS--LSGT-----------SSVLETQYFPPIELDSDKMYVLGLVELLTFNSIP-----------NV   48
P-6_NVi_PX          1  ME--------------NS-LTLT--LSGS-----------SSILEAQYFPPIELSPHKQYVLGLVELLTFNSIP-----------NI   48
P-2_TC_PX           1  MQII---------SLVESKMLFV--LTGK-----------SAILSADFNPPIDVSD-GVYELGVTNFEVYNSIP-----------NI   53
P-1_CI_PX           1  MV--------------ED-FYVT--LPSNAS-LEYFPNNTLTNYVTRLPKEFHLQ--GTWVVGLTEVAFPYSWY-----------TI   56
P-2_CI_PX           1  MT--------------QD-FYIT--LPSNAS-LNYFPNNTLTNYVTRLSKSINLE--GTWVVGLSEVTFPFSWY----------NVS   57
P-5_SP_PX           1  MA--------------SH-FYVS--LPSNSS-MLYYPDNKTCKYTTKLHAPLSLH--DDYEVGLSEIQFQCTWY-----------NV   56
P-3_SP_PX           1  MG--------------SH-FYMT--LPSNSS-MQYYPNNKTCKFTTKLHTPLSLN--GDYEVALVEIQFQCTWY-----------NV   56
P-1_SP_PX           1  ME-------------SEDQVYLT--LPSNSS-FDYFPNNTLTSFTTKLATPLVLR--GEYEVALVDIIYPHSWT-----------NV   58
P-2_SP_PX           1  ME-------------SEDQVYVT--LPSNSS-FDYFPNNTLTSFTTKLATPLVLR--GEYEVALVDVIYPHSWS-----------NV   58
P-2_XT_PX           1  ME-------------EEA-FYIT--LPSNAS-LSTFPQNEISNYTVKLSKPVMLR--GEWEVGLTEIQYPHTWN-----------TF   57
P-1_XT_PX           1  MD-------------EGS-FYMT--LPSNAS-SKIYPD------TTKLAKSVDLR--GPWEVALTEIQYPHTWN-----------TF   51
P-1_CPB_PX          1  MS-------------DGG-FYIT--LPSNAS-SAVFPQNTISNFTIRLIKPLDLP--GAWEVGLAEIQYPHSWN-----------TI   57
P-N1A_NV_PX         1  MF-------------NTM-VVLH--AVFT-----------GGELTLFFEQPIR----RPRFVALRQCSLFNSWY-----------NL   45
P-N1B_NV_PX         1  ------------------MVVLH--TVLT-----------KGEQTLFFERPIR----RPRFVALRQCSLFNSWY-----------NL   41
Consensus_aa:          M...............p..hhls..h.ss............s.hphph..sl.lp....bltl.ph.h..s..............sh
Consensus_ss:                           e eeee  e                eeee     eee     eeeeeeeee     e

Sputnik_V18-V19    79  NVN--NTIYSVSIGYN---GTYSSQNFVQFDPSLTSPNIPAPNAPTVTSPNVEVTPYYYIYDYSTFLQMINTALENAFNEISAPVGA  160
Mavirus_MV17       62  FKPA-GHDYQVGIFNR---STDEIMGVREVDFS------------------TFVDEDGYMYDYVDVGTAINETLAGLCDG--IIGEE  124
Tlr_penton         48  NQY--NNTSQINSK------TYSI-------------------------------PIGI-YDVPSLINQIKSLVTP-----------   83
P_AP_PX            48  NPT--NNRLKIETIPPKRKKDDYHVFEFCL-------------------------EDGC-YEIEDINNYMAKELSQVNN-----DHG  101
P-1_NV_PX          61  PEYG-NDSVKYSPDG---GATWTTVTF----------------------------PSGM-YSYHDLNDFLHQDMKSNGHVG-VADGK  113
P-4_NV_PX          62  DST--NNNFRYSPDN---GATWFNIDI----------------------------PEGS-YEITDINDYVQRIMKDNGHY---DSAN  111
P-4_NVi_PX         49  DTG--NNKFYVGG---------EVIIL----------------------------PTGS-YEIEDIEKSLKEALTP-----------   84
P-3_NVi_PX         49  DFK--SNKFYVGQ---------EIIEL----------------------------PTGS-YEIEDIERTLQEILSL-----------   84
P-6_NVi_PX         49  DKG--NNKFYVGK---------EEIVL----------------------------PTGS-YKIQDIDSXLREILTK-----------   84
P-2_TC_PX          54  DEE--NNKFFFGD---------VEFKI----------------------------PTGC-YQLTDINNYLQHVIEKQFS-------N   93
P-1_CI_PX          57  PQQ--AVTMKIRRGP---DLGVIEADI----------------------------SPGYYNSPKELIESIVKLFTDVGY-------A  103
P-2_CI_PX          58  KKASRGLKVKQRVG------GITGIDI----------------------------SPGYYESPRLLINNIASTLRGVNF-------T  103
P-5_SP_PX          57  RKG--NNTLYIY------DRETVTDSVPLLR--------------------QITVQEGYYMNGVDFIHAINAALDVAT--------R  107
P-3_SP_PX          57  REI--NNTITVF------NKNT-EDGVPLVR--------------------NVTLPTGYYYTGEELITMINASLDQDW--------R  106
P-1_SP_PX          59  NFT--NNKYSFSIG----DQVITVGRV----------------------------PVGHYRDAQSVCTAFNESLPANL--------R  103
P-2_SP_PX          59  NFT--NNKYSLSIT----DQVITVGRI----------------------------PEGHYREALSICTAFNQSLPPHL--------R  103
P-2_XT_PX          58  ETD--EGLFYVGIH----GGPLKELNV----------------------------KPGLYNSVKDLVKAINDKIEAYK--------S  102
P-1_XT_PX          52  DPH--EGNFVVGKQ----DDLLKEYHI----------------------------KSGYYNTINEVVKAINARLDSLK--------I   96
P-1_CPB_PX         58  NED---TPFEITFG----ATTWN-FIL----------------------------RRGYYSTIPELLEHMNSNVARHPG-------P  101
P-N1A_NV_PX        46  ERE--HQITTIASL-------PPIVTL----------------------------PAGH-HTAESTVETINHVKSQ-----------   83
P-N1B_NV_PX        42  ERE--HQITTIAAL-------PPIATL----------------------------PAGH-HTAESIVETINRAESK-----------   79
Consensus_aa:          p....sp.h.h..............pl..............................Gh..ph.sl.phlpp.h.............
Consensus_ss:                 eeeeee      eeeeeee                                   hhhhhhhhhh

Sputnik_V18-V19   161  DAPFFFYDSNTEKISLIAQAAYYDRTLTTPIEIYCNVNLFTFFDSIKHIGLGYNTPTGRDILFDVRFLGNNYYQDPETAPSYPPEFI  249
Mavirus_MV17      125  DIPVFSFNKHSKKFEITTTENFRN-----GHFIMFNDDMRVDF--------NSF---------------------------------  165
Tlr_penton         84  DLSSFLVQFSSLTYRITFSASTN-------FTMSFNQSLANIL--------GFQ---------QTQFQSNSSY----TTTK------  136
P_AP_PX           102  THLTFSIRMDPVDFRTYIKCNGI-------LRFNTSFSIAPVF--------GYR---------KKDCGPFHE----EHRSD------  154
P-1_NV_PX         114  DKFYIDIVFVLTTYKVVVIVGDKYE-----LDIR-GTKFGELI--------GFD---------PKVISKTEY------GSK------  165
P-4_NV_PX         112  DEYYITIEPNNNTLKSVVDISANYK-----VDSTTANSIRTVL--------GFN---------SQVYSEGYN---------------  161
P-4_NVi_PX         85  KGITLKLKPNNNTLRCMIKCNRS-------IDFQPDDSIGKLL--------GFT---------SRVLSPN--------TDY------  133
P-3_NVi_PX         85  KNITLIIKPNNNTLRSVITCSHE-------VDFRPKDSIARLL--------GFT---------PRLLKPY--------ITH------  133
P-6_NVi_PX         85  KKISISIQPNNNTLRSIIKCNRK-------IDFRPQDSIGALL--------GFT---------QRVLQEN--------XKH------  133
P-2_TC_PX          94  DLLSITANNNTLHTHIKATKD---------VDFTKPNTIGPVL--------GFN---------SQIVPKN--------IGK------  140
P-1_CI_PX         104  NNIEVSYNDLTGKVRVNVHKKGW-------YLEF-SDVLSRML--------GYD---------KKRLRRGSH-----EAER------  154
P-2_CI_PX         104  DYIQISYIPITGKASVAV-AKGW-------YFEV-TEPLKQML--------GFR---------KKRLGYGYH-----ESDK------  153
P-5_SP_PX         108  EKVNFSYIEASRKCAIQVQPGIG-------IVL--PCSLARML--------GFF---------EKCNLTQST-----ESPS------  157
P-3_SP_PX         107  QRVGLFYKKSSRKVYVQLRADTG-------LLI--PCNLARML--------GFD---------RECHLLEST-----ESPM------  156
P-1_SP_PX         104  NKASFNINPSTLKVRAEIQPDSG-------VYL--SEGLGQLF--------GFP---------EGTLCTNQ------EGKF------  152
P-2_SP_PX         104  NKACFTINPSTLKVRATIQPDTG-------IYL--SEGLGQLL--------GFP---------EGTLRANQ------EAKF------  152
P-2_XT_PX         103  PTYDVKLRYDELERIVTVKGTHS-------FLA--GNKLTHIL--------GID---------SNNFNDSI------NGQL------  151
P-1_XT_PX          97  PHEHIKLRYDDLERSVSVSESPI-------YTFAPGEKLAHIL--------GMD---------GYIAPYGTSL--PKVKKI------  151
P-1_CPB_PX        102  PEVVMNYDPVGRKVRLKSTDFMY-------VFS-TDGELANIL--------GLG-----------HKRNVQ------KFPF------  149
P-N1A_NV_PX        84  -ILTAKLDPLTGKIVLVSTGI---------VFL--NAELCALF--------GLE---------AKDDQMGE------RGLA------  129
P-N1B_NV_PX        80  -ILTAELDPLTGKIALMSNGV---------AFL--NAELCALF--------GLE---------AKDDQLGE------RGLA------  125
Consensus_aa:          s...h.hp..s.ch.h.hp...........h.h..s.pl..hh........Gh.............h..sp................
Consensus_ss:            eeeee    eeeeeee    e       eee      hhh
```

```
Sputnik_V18-V19   250  QMQQEYPT--LSNWNAVKTIQLVSNLLPI------------NKESIPSFRNSNVGIINAQGILADFVPLVTNGPEARISIDFVATGP-  320
Mavirus_MV17      166  --EFD-------DIDEEYSLVILNEDVETQDASTLEFLTPISHIVIESNDLPVSYELLPSISK-------------NTTISDNTGVFL  231
Tlr_penton        137  --TPS-------INSPQTILINIKDIPD-------------SQIIQAQNYD-VDFSFMILNISNR-----------GQNIQYENRSD-  189
P_AP_PX           155  --KAT-------NLNTINSIKVMCNIAH--------------GSFNNQLQSHSIYEFFPSGRT-------------GTKVVQSPVNL-  205
P-1_NV_PX         166  --LPN-------ITNSIDTLHINTDLIT--------------DSIVGGRASNTLFVIPTDN----------------LS--RSYPFSV  212
P-4_NV_PX         162  --ESEN----IVNILSVNSLRVTSDVIA--------------SSYTNGGTENIIYSFFPSVGP-------------GYKIVQEPLNL-  215
P-4_NVi_PX        134  --ESDL----PVTILKINALRVECNITS--------------GAYINEHKVHTIHEFFPAVSP-------------GYKIIEVPSPI-  187
P-3_NVi_PX        134  --NSDL----PVAILKVNALRVECNITS--------------GAYINQNQVHTIHEFFPAVPP-------------GYKIIEVPKQI-  187
P-6_NVi_PX        134  --SSDL----PVAILKVNALRIECNITS--------------GAYINGQLAHTIHEFFPAVPP-------------GYKIIEVPSQV-  187
P-2_TC_PX         141  --DSDN----IADIMKLNSIMIECNITI--------------GSFKNGEPVHIIYQFFPNVPP-------------GFKIVQSPDHV-  194
P-1_CI_PX         155  --VCD-------ISLDMSLLYFYSDIVQ--------------GRSVGDTLAPLLAIVPVKGRP-------------REAVYQRFDYP-  205
P-2_CI_PX         154  --VCD-------VNLDLSTLYIYSDIVE--------------GRAVGDTLAPLLGIVSVKGKY-------------GKAIYQRFDKP-  204
P-5_SP_PX         158  --PVD-------VHMYFHSFYVYSDILQ--------------FRHVGDTSVPLLRTIAVMPRSR------------QENIVNTYIAP-  209
P-3_SP_PX         157  --PVD-------PHVEFHTFYMYSDIVQ--------------YQHVGEVSVPLLRTIATKARKD------------QYNSVSTYNTP-  208
P-1_SP_PX         153  --LPD-------LNGGLFAMYVYTDIIE--------------NQRVGDISAPLLRIVAVDHKRA------------GEVVDRSYQTP-  204
P-2_SP_PX         153  --LPD-------INGGLFAMYVYTDIIE--------------NQRVGDTSAPLLRIVAVDHQRA------------GEVVERTYQSP-  204
P-2_XT_PX         152  --CAD-------IKAGFYTLFVYTDIIR--------------PQRIGEFYTPLLRTVPITGSN-------------NEIVTQQFIKP-  202
P-1_XT_PX         152  --YAD-------IKAGFYTMFVYSDIIQ--------------HQLVGDSYVQLLRTVEISGKN-------------NEIITQRYTRP-  202
P-1_CPB_PX        150  --SAD-------ITGGFNSLYLYTDIVE--------------HQFVGDFSVPLLRCVPVRGRN-------------NEFVTITYDKP-  200
P-N1A_NV_PX       130  --LPLGDPPLKVTLPKIEAIYIFCDIVDR------------SQCLAFDEPSNVLACLETRGRP-------------HEKVVYGPDIP-  189
P-N1B_NV_PX       126  --LPLGEPALKVALPKIEALYICCDIVDR------------TQCLSLGEPSNVLACPETRGRP-------------HEKVVYGPDVP-  185
Consensus_aa:          ...ss...........h.sl.l.sslhp.................h....s..l..h.sps................b.l........
Consensus_ss:                          eeeeeeeee                  eee         eeeeee                    eeee

Sputnik_V18-V19   321  -----WRLIDMFGSVP--------------------IYMVDLYVYWTDQTGGQYLINI-PPGRILTCKLVFIKKSLSKYLVSEK  378
Mavirus_MV17      232  --TNYKYLQQNNQDYNSILFRVENSSNKYHNILQTNFNRFNLSFTIYDYDNEKHPLTLLPQTV-IQLKLLFESID---------  303
Tlr_penton        190  -----ENTIEITG-----------------------DIRQISVQLYKNDGQ---FFM-INSG-IQLSFQYE------------  227
P_AP_PX           206  -----IYYRLNKT-----------------------DINSITVQLVDQNNN--PIDN-FNET-LTVVLHIKRHGSDH------  250
P-1_NV_PX         213  QPTRALFNNVAAN-----------------------LISSMRFYVVDSIGR--PINL-NGIE-WHMTLILRSIY---------  259
P-4_NV_PX         216  -----IYLPITLG-----------------------TISQMETKLVDQNGK--LINL-RGEE-LSIRLHIREA----------  256
P-4_NVi_PX        188  -----IYLPVTVK-----------------------TINNLQLHIVDQDGH--LVNF-RGEV-ITIRLHIKSV----------  228
P-3_NVi_PX        188  -----IYLPITVK-----------------------IIDHLQLRIVDQDGD--LVNL-RGEV-ITIRLHLKNV----------  228
P-6_NVi_PX        188  -----IYLPITVK-----------------------SIDHLQIRIVDQDGH--LVNF-RNET-ITIRLHLKPT----------  228
P-2_TC_PX         195  -----IYLPISVK-----------------------TIRNITLKIIDQDEK--LVNF-QQET-VTVGLHLQKKEENGY-----  240
P-1_CI_PX         206  -----CYHPIQNK-----------------------VFHTIEVDIRDSLGK--KILF-QRGR-VCVTLHFKRVNKA-------  249
P-2_CI_PX         205  -----SYHPIQNK-----------------------NFHTIEIDLRDSTGD--PILF-ERGR-VCVTLHFKRVNKI-------  248
P-5_SP_PX         210  -----HYLPLKLF-----------------------NFETIDIIITNETGE--VVPF-ERGK-VLVTLHFRERSSRLS-----  255
P-3_SP_PX         209  -----HYVPLKLY-----------------------NFETIDIILTTESGE--VVPF-ERGK-LIVKLHFRERSPSL------  253
P-1_SP_PX         205  -----HYLPVKSK-----------------------YIDTIHIDIRSDFGD--KVPF-QNGK-VVLKLHFRSVRRRSFHL---  252
P-2_SP_PX         205  -----HYIPVKRK-----------------------YIDSIHIDIRSDFGD--KVPF-QNGK-VVLKLHFRSLRRPTFGF---  252
P-2_XT_PX         203  -----DYLPVSKH-----------------------HFDNITIEIKSDQNR--NVSF-KYGK-AIVKLHFRPRRAYY------  247
P-1_XT_PX         203  -----DYIPVCKQ-----------------------HFDSVAISIYSDQCK--PVKF-KYGK-CLVRLHFRPRKELSY-----  248
P-1_CPB_PX        201  -----HYVPVSKH-----------------------HIDTITIEIKTDQNR--HVSF-RFGK-VIVKLHLRPRRERGF-----  246
P-N1A_NV_PX       190  -----ICVAASSS----------------------EFVSSIRIWIRDDRGR--RVDF-KDKP-VRLVLELT------------  229
P-N1B_NV_PX       186  -----VCVAASSS----------------------EFVSSIQIWIRDDCGR--RVDF-KGKP-VRLVLELT------------  225
Consensus_aa:          ......hh..............................hpslpl.l.sp.sp...l.h......l.lpLhhpp...........
Consensus_ss:                eeee                            eeeeeeeee        ee       eeeeeeee
```

Figure S2. Multiple sequence alignment of virophage penton base proteins with the PX proteins from Polinton and Tlr elements. The last two lines in each block show consensus amino acid sequence (Consensus_aa) and consensus predicted secondary structures (Consensus_ss). Representative sequences have magenta names and they are colored according to predicted secondary structures (red: alpha-helix, blue: beta-strand). If the sequences are in aligned order, the sequences with black names directly under a representative sequence are in the same pre-aligned group. Consensus predicted secondary structure symbols: alpha-helix: h; beta-strand: e. Consensus amino acid symbols are: conserved amino acids are in uppercase letters; aliphatic (I, V, L): l; aromatic (Y, H, W, F): @; hydrophobic (W, F, Y, M, L, I, V, A, C, T, H): h; alcohol (S, T): o; polar residues (D, E, H, K, N, Q, R, S, T): p; tiny (A, G, C, S): t; small (A, G, C, S, V, N, D, T, P): s; bulky residues (E, F, I, K, L, M, Q, R, W, Y): b; positively charged (K, R, H): +; negatively charged (D, E): -; charged (D, E, K, R, H): c. The alignment was constructed with PROMALS3D (http://prodata.swmed.edu/promals3d). Abbreviations: AP, *Acyrtosiphon pisum*; NV, *Nematostella vectensis*; NVi, *Nasonia vitripennis*; TC, *Tribolium castaneum*; CI, *Ciona intestinalis*; SP, *Strongylocentrotus purpuratus*; XT, *Xenopus tropicalis*; CPB, *Chrysemys picta bellii*.
