# Supplementary material for: Conservation of major and minor jelly-roll capsid proteins in Polinton (Maverick) transposons suggests that they are bona fide viruses
Source: Biol Direct. 2014 Apr 29;9:6. doi: 10.1186/1745-6150-9-6 (PMC4028283; doi:10.1186/1745-6150-9-6)
Supplement: Additional file 4: Figure S3 — Multiple sequence alignment of the major capsid proteins of PBCV-1 and Polinton 3 of Nematostella vectensis (P3_NV) with the protein 00012 of the virophage PgVV. The last two lines in each block show consensus amino acid sequence (Consensus_aa) and consensus predicted secondary structures (Consensus_ss). The protein sequences are colored according to predicted secondary structures (red: alpha-helix, blue: beta-strand). Consensus predicted secondary structure symbols: alpha-helix: h; beta-strand: e. Consensus amino acid symbols are: conserved amino acids are in uppercase letters; aliphatic (I, V, L): l; aromatic (Y, H, W, F): @; hydrophobic (W, F, Y, M, L, I, V, A, C, T, H): h; alcohol (S, T): o; polar residues (D, E, H, K, N, Q, R, S, T): p; tiny (A, G, C, S): t; small (A, G, C, S, V, N, D, T, P): s; bulky residues (E, F, I, K, L, M, Q, R, W, Y): b; positively charged (K, R, H): +; negatively charged (D, E): −; charged (D, E, K, R, H): c. The alignment was constructed with PROMALS3D (http://prodata.swmed.edu/promals3d). [file 1745-6150-9-6-S4.pdf]

```
PBCV1_VP54        1  MAGGLSQLVAYGAQDVYLTGNPQITFFKTVYRRYT----NFAIE-SIQQTI--NGSVGFGNKVSTQISRN  63
P3_NV             1  MAQAISEKI-----------NP---------NRIPREPFGLKAESSLNRITFNPSSASPGETLYINIPKL  50
PgVV_00012        1  M-----------------------------MDRIS----GFSTN-SYRLQVLGSSSASANQIVRFELPSN  36
Consensus_aa:        ...............................pRhs....sh.hp.Shp..h..stSht.sp.l.hplsp.
Consensus_ss:           hh h                                       eeeeee         eeeee

PBCV1_VP54       64  GD----LITDIVVEFVLTKGG--NGGTTYYPAEELLQDVELEIGGQRIDK-HYNDW--FRTYDALFRMND  124
P3_NV            51  AENVVIVPGSVSLLFDLNVTGHANNTLVNNVGRNLVSRLKILFGGETLQDTQRYDL--FQTYHDLYLQAE  118
PgVV_00012       37  S---IVDIRKFAMAFKLAVTG--DDHARLSDVNNIVERVSITVGGTELSS-GFSKYNVLKQIKKCLM---  97
Consensus_aa:        t........phsh.F.Ls.sG..ss.h...ssppllpclpl.hGGpplpp....ch..hpphc.hh....
Consensus_ss:            eee  eeeeeeeeeee       hhhhhhhheeeeeee           hhh  hhhhhhhh

PBCV1_VP54      125  DRY----------NYRRMTDWVNNELVG----------AQKRFYVPLIFF-------------FNQTPGL  161
P3_NV           119  DREDRIKQGISSENMRKLRTNAGDKATSDAKEVALAAVHNTKYCIPLDHP-------------ILGEHGV  175
PgVV_00012       98  ------------------ADE-GCVLTE-HPEIARTKSYNTGVAYNLNGGEPAADYRIDEWMGF---LGE  144
Consensus_aa:        ...................s..ss.hh...........hpp.hhhsL................h....G.
Consensus_ss:         hh          hhhhh                        eeee                     h

PBCV1_VP54      162  ALPLIA--LQY-HEVKLYFTLASQVQGVNYNGSSAIAG---------AAQPTMSVWVDYIFLD-------  212
P3_NV           176  FYP-----KALPHPLIFEITLAPVSDVVVYADTVKTP-----------TYTITNLELEYACIS-------  222
PgVV_00012      145  CEPRVIDTSKF-PPIQISITLAAAQAVVVQGSLAGTPAQFVTEGSTNGDYSLTDIYATVPVLAFNDGLYD  213
Consensus_aa:        hbP.......h...lbh.hTLAs...sVsbss.s.hs...........sbshhslbhph.hls.......
Consensus_ss:        e  e          eeeeeee    eeeeee                  eeeeeeeeeeeee

PBCV1_VP54      213  -TQ--ERTRFA--QLPHEYLIEQLQFTGSETATPSATTQASQNIRLNFNHPTKYLAWNFNNPTNYGQYTA  277
P3_NV           223  -SEYLAREALSAYQVGRGFFYENVIL---------------------------HKTFTISKP-NDGVINE  263
PgVV_00012      214  GMT--EAILQK--QGFLELPYKNYIN---------------------FQNVGNSVRWNCSSASIDRIWTA  258
Consensus_aa:        ..p......b...Q....h.hcp.................................@shspss....hs.
Consensus_ss:         hh  hhhhhh     ee eeeeee                            eeeeee       eehh

PBCV1_VP54      278  LANIPGACSGAGTAAATVTTPDYGNTG-------------------TYNEQ--LAVLDS-AKIQLNGQDR  325
P3_NV           264  HINLPRRSMT---GILCLFTESYTGGA-------------------RDSEKFVNPSITS-ININVDGMPN  310
PgVV_00012      259  TRDLTHNSKK-----APVLVEGYNGDYGKMFNYADEKYQSRYMNFPSPSSS---AGITSQHRINSSLLPQ  320
Consensus_aa:        h.sls..t.......hslhh.sYsss....................p.spp...sslsS.hpIp.s.bsp
Consensus_ss:        h             eeeeeee                                   eee eeee     h

PBCV1_VP54      326  FA------TRKGSYFNK--VQPYQS-IGGV----TPA---GVYLYSFALKPAGRQPSGT-------CNFS  372
P3_NV           311  RLYSKGMTPPDL----WESVKKRFG-REGVKQ--KDFYANNKFALWVDLRA---HPDNSIHGGGLVLNNT  370
PgVV_00012      321  FA----MSPMDSALLTKQSVPKHYQKKHGLKTMTSNF---NASCVRLNLEG---SEQLR-------IASG  373
Consensus_aa:        .h......s.c.....b..V...b....Gl....psh...s..hh.hsLcs...p.p.p.......hs.s
Consensus_ss:        hh       hhh    hh  hhhhh h              eeeeeeee                 e

PBCV1_VP54      373  RID--NATLSLTYKTCSIDATSPAAVLGNTETVTANTATLLTALNIYAKNYNVLRIMS-GMGGLAYAN  437
P3_NV           371  R-D--GVKLEMKRKVGGT--GNITCYMF-VVA----DAL--------------MEVMNSNLRAIMY--  412
PgVV_00012      374  L-DSRSIALQGHFDLNGLDSNDTPIDVF-VET----TSVLRVGRNL------MIEVVA----------  419
Consensus_aa:        ..D..sh.Lp.p.chsth..ss.sh.h..h.h....sth..............hclhs..........
Consensus_ss:             eeeeeeee           eee   ee    hhh     ee      eeeee    hhh
```

Figure S3. Multiple sequence alignment of the major capsid proteins of PBCV-1 and Polinton 3 of *Nematostella vectensis* (P3_NV) with the protein 00012 of the virophage PgVV. The last two lines in each block show consensus amino acid sequence (Consensus_aa) and consensus predicted secondary structures (Consensus_ss). The protein sequences are colored according to predicted secondary structures (red: alpha-helix, blue: beta-strand). Consensus predicted secondary structure symbols: alpha-helix: h; beta-strand: e. Consensus amino acid symbols are: conserved amino acids are in uppercase letters; aliphatic (I, V, L): l; aromatic (Y, H, W, F): @; hydrophobic (W, F, Y, M, L, I, V, A, C, T, H): h; alcohol (S, T): o; polar residues (D, E, H, K, N, Q, R, S, T): p; tiny (A, G, C, S): t; small (A, G, C, S, V, N, D, T, P): s; bulky residues (E, F, I, K, L, M, Q, R, W, Y): b; positively charged (K, R, H): +; negatively charged (D, E): -; charged (D, E, K, R, H): c. The alignment was constructed with PROMALS3D (http://prodata.swmed.edu/promals3d).
