# Supplementary material for: Conservation of major and minor jelly-roll capsid proteins in Polinton (Maverick) transposons suggests that they are bona fide viruses
Source: Biol Direct. 2014 Apr 29;9:6. doi: 10.1186/1745-6150-9-6 (PMC4028283; doi:10.1186/1745-6150-9-6)
Supplement: Additional file 5 — Fasta-formatted sequences of the predicted major and minor capsid proteins of polintoviruses discussed in this study. [file 1745-6150-9-6-S5.doc]

**Major capsid proteins (PY)**

>Tlr_4Fp'

MDELLRTMKIDRIFIFLFDNIIQQRKIRLQMSQLLYHVQITNYLASAYSFNTIYNLINYI

EIFNDNKSVEKLDSQTLFMSNLLQVKQDTYQLQNSLKGFNPTTLAANNGYAEFLIDINCF

LTKQAIPMSSLGDIRIDIKFNADTATASIDNVQLTVSEVLVFYYEITDSQDKYFRGLKCI

DNRYLQINHASYNWNNITANYSFKFNLTQDAVIPFMLFYIQNKSDNDSFTTTYQNLVPVI

AFELLGKNNQSTMNNVVMTQTYNLFQIQKHFPQQFHIVNPSSNYYNNVYMLNFCLNPELA

MKRNFVGGQEISDNDYQLKITVGQNISQAVIHVFLFNYQMTRIERKKVIFFDQ

>Polinton-1_AMi

MAFVHCGSEECAKSELDVFQIAPTQTCIEKSLYVEIPPLSAITESGPLDFFIAGNGEDYM

DLNNTLLYLCCKIVKGDGTDLDAGAAVGLVNYPLASVFSQLDVTLGDHLISQSNNCYPYR

AFIESVLNYSDDTLATQFSAGLFYKDTPGHHETVARDGDNHGFRRRANLTDRSKKIELLG

HLHSDLFFQEKLLLNGVDVKIKLTRSKDAFCLMGAAAAGQCKLKITSASLFVKKVKVAPG

VRLGHAEALLTANAKYPIDRVGMKVFSIPAGSRVSNQENLFLGQLPKLIVIGFVDNDAFS

GNYAKNPFNFKHYNINFVALYVDGEQTPTKPLQPDFENGNCVREYMQLVQTAGKHMKDRA

LIINREEFALGYTLFAFDLSPDQECADHYSLIKTGNLRAEIRFAVALPTTVNMIVYGVFD

NVIEINQRRNVLFDYM

>Polinton-N1A_NV

MSVSDKLDPQRTPRIPLGLKAERTVHRVTLNPSTASPGETLYMAVPKLSEGVTLVPGSLR

VGLDLSVSGHANNTVVKNVGRNLVSRLKITFAGETLQDTNRYDVFKAYEELYLSKVERED

RLEQGIQSENMRKLRSEAGDKATSNAKENALSAAHGARYTIPLDHSLLTDHGVLYPRALS

EALLFEITFASVDQVVVGTDATKLSYGIKNLELEYESLRDDNLARSAAAAYHNGTTFFYE

QVNLHKTFVISKGTDSIINESINLPRRSMSGLLLLFVEPYTAGTRDSEKFVYSDIKSVRV

TIDGMPNKVFSQGLRPTDFWREAKRRFVRPYQGGHSGAPAAPVIGPGSFYGDNKFALWID

LRTTDDSAIHGGGLRLVNTRDGVHLEIKRTAGGSGSVNCHVFVVADAQMNLMNGQLESIQ

Y

>Polinton-N1_NV

MSVSDKLDPQRTPRTPLGLKAERTVHRVTLNPSTASPGETLYVAVPKLSEGVTLVPGSLR

VGLDLSISGHANNTVVNNVGRNLVSRLKITFAGETLQDTNRYDVFKTYEELYLSKVERED

RLEQGIQSENMRKLRSKAGDKATSNAKENALNAAHGARYTIPLDHSLLTDHGVLYPRALS

EALLFEITFATVDQVVVGSDATKLSYGIKNLELEYESLRDDNLARSAAAAYQNGTTFFYE

QVNLHKTFVISKGTDSIINESVNLPRRSMSGLLLLFVEPYTAGARDSEKFVYPDIKSVRV

TIDGVPNRVFSQGLRPTDFWREAKRRFVRPSPGGHSGAPAAPAIGPESFYGDNKFALWID

LRTTDDSAIHGGGLRLVNTRDGVHLEIKRTASGTGTVNCHVFVVADAQMNLMNGQLESIQ

Y

>Polinton-6_NVi

MDEILSIQSPVSFDESLAHYELHAHQPYTVSSYNNSDEIRIAIQHQDLSLLPSRSSLHVC

GKLTKPNGTALARTKLVNNAICHMFEEIRYEINAVEIDKCKNVGLTTVMKGWISHNPSQS

LIMENAGWLDIAETKSLTNASGYFDVNIPLSMIFGFAEDYRKIVVNVKHELVLTRSRNDL

NAIIQTATLADGVATFEEYKLELTKIEWLMPYVVASNTNKIRLLNYIEKNRPISMSFRSW

ELYEYPVLPTSTKNVWTVKTSNQLEKPRFVILGFQTNRKNQQAENASQFDHCDISNVKLF

LNSQYYPYGNLNLDINRYQYAVLYDMFANFQSLYYDKVSEPVVNKNDFISRLPLIVIDCS

KQNESLKSAPVDVRLEFESRDNFPAGTSAYCLILHDRIVQYNPVSGDIKTLI

>Polinton-5_NVi

MAFLHSHSTECMSSELDLFTLPATQTSIESSSFLHYKPVSSLSDDVDAPLEFVVPAGSEH

YFDLAHTMLHVQAKIVPADEATATTEDLKVGPINNFMHSMFNQIDVFFNQKIVSPPNNAY

PYRAYIETLLNYAPAAKESHLTASLWYDDTSGGFDSPANAVSTATAPMIVNKGLENRKYF

TQNRRYFDMIGHLHHDLFNQDKMLINGVEMRVRLVRSKDAFCLMDATADGKFKLSIKEAT

LIVRRVKISPGVLLAHAQALSKTTAKYPITRVEVKSFTLHSGILGDSIDNVIHGQLPKRI

ILGFVENKAFNGNRALNPFNFQHFSINYISLYVDGVQIPSKPLQPRFTGLDKLYIDAFQT

LYTGTGVHFLNEGFGINRYNYYKGNFLTAFDLTPDLSAHCATHWNLVRSGSIRIEVRFET

ALLTAINCIVYAEYDNVLEIDSSRQIVTDFSA

>Polinton-5_NV

MSVFTGSXQVANXGMBLFTLPPTDVALANGHYIPYTPXDEGDNHIEWYISXSSTYIDLKK

THLRLEVKITKGDGSDLAAGEKVGFVNNALHSMIKQVKVELNNRPVTLQSDSYAYRAYIE

SLFNYSKEAQXSYLTGALWYKDTAGQHEXTAVASSGSGVATNQGLXTRAAFTAESATVGL

TGPLFCDIFHSERYLLDQIPIKIRIELQKPQFALMSQGENXLLXNSKFPREQFXACTTP

PPTXSGITRRKCFPLKHNLAKYPLTTAVVKTGQIXSGKRSILFRNLFNGLVPKRMIFGI

VEDAAYNGDYTKNPFNFQMPQSEXYRGQSGWKRNTYIXSLKNIDGEGVDLYHLLFDNVCG

XYRGRGLNITREEFKXGYALFPFDFTGAANASSGYFQPQYKGVVTLELSFNXATDKVYTV

VLYGEFNNNMEIDPARNVMYDIQG

>Polnton_GlyFla

MAEILNINKSIIFDESIAHCEAHSHLPYASSTFNNSDEIRIAVQHQDLCLLPSKSTLHLY

GRMRKADGTAVSATTKMVNMAVCHMFEEIRYELNGVEIDRCKNVGITSLMKGYASVSPGQ

QNILENAGWIVNEDDNKLTDNGGYFDVSIPLSFILGFAEDYHRIIMNAKHELILIRSNTD

TNACTVSAAAADDVAITIQKIEWIVPYITMSDKQKIEALNYITSDPAIPISFRSWELYEY

PLLPATSKHIWAIKTANQLEKPRFAILGFQTARKNQPDQNASEFDHCNITDVKLFLNSQS

YPYGNLNLNFAHNQYALMYDMYKNFQVAYYGKEAEPLLTKKKFLEISPLYIIDCSKQNES

IKSGPVDIRLEFESTAPYPIIIHQLTV

>Polinton-4_SP

MALLHEKSDICCKSELDLFSVPYTQMSIVKGPWVEYHPVSNITDSGPIEFNLSGTAEEYM

DLSQTMVHVLAKVVNPDNTDLAADAPVGPINLFLPSLFSQIDVKLNETLVSQPSNTNAYR

AFIDVLTHYGNDTMSSQLTQQLFYKDNAGKLDAVNPLLPEGEVNHGLKKRHGFTSESKTV

SLLGPLYGDIFFQERLMLPGVDVNIKLNRTKDAFCLLTSHPTLKYKVMIEKATLYVRKVK

VNPSVMLSHAKGLESSPAKYPINKVDVRSFTIPAGNMSLNKDNLFLGQIPNRIIIGFVDN

DAYNGSYRKNPYNFKHYNLNYISITVDGEPLPMRPMRPCFEEGKSQNYIEAYNTLFLGTS

RLFSDRGIAINREEYAQGYTLYAFDLTPDLSDGCHLNLIKEGNVRLEAGFDVALPNTLNC

IVYSESQGLIQIDRSRNVINDFKG

>Polinton_CB

MIEAVDKMLVVDTPIVSDTSIQKENLISIFPSSGSSLNQHGEINFVIETCDQYLHLHKSY

IYMECELLKSDDTALAEADEVSLTNNAPMFLFDRSTYSMDGMQVESIMDPGRASLMKGLL

TYSSTMNKQNTFGWILETKDIITYKINKYTKNRKGSITFCIPLTHIFGFAECYNKIVYGR

KHQLTLYRSPENKNSITSNDAKKHTAKINIKKLQWIIPKIVPSLEMQNKLLNIFESSKLV

QMPFISRQLESYNIPNGNREFTWKLGTKYDKIKYIVVALQSNRDNNYLNAGKFDNCGLEE

IYVELNSERYPHDCLKFDFDKFNAVQQYNFAKEFRNSYYESTKDYIFMEEDVYYYYYPLL

VFDVSKQNDRIIQSRPDVTIKASFSKSIVQNTKCYCLIMSENIVEIKDNRVKVVSV

>Polinton_Od1.2

MDRPDRKIIKKHYPRENLDERLSFTFEADPNLCLVKNKIQIFFTIEVDEKYVPENGFAAK

QFSCIDVELNSMLVSTTKTRGQYYMNDWLLKKINFTRDYLYSMFRIEGYFDDYNYEDLAD

DDKIKIAKTRRSGCLLKDKKYIYQLCMIPTDAFLLDNKPLPPNLEMKLTLNRQNKKFSCI

NTGEADLFPAPFVLKDCYANVEYISSSNLRQYLDPKRPISYKYDELKMITRVLPRGERRL

RIENLAGGNTPEYLFVGLIETGLLGGDDKKESINFLQHGAIQFDLLLNGSSCNGYPMAIQ

SQYPLKPYLKYQDVLGNILNTKSPMVDSIFDFKKALLYAHEFQGDIEDENGWISVAIDFK

EPVDSNLSIVLWTVDMVRIAIDEYGQIEKMLL

>Polinton_CE

MIEAVDKILMVDNPIVSDTSIQKENLISIFPSSGSSLNQHGEINFVIETCDQYLLLHKSY

IYMEVQISKLDDTALAETDEVSLTNNAAMFLFDRATYNMDGMPVESIMDPGRASLMKGLL

TYSSTMNKQNTFGWILETKDIMTYKINKYTKNRKGNITFCIPLTHIFGFVECYNKIIYGR

KHNLSLYRSPENKNSITSSNPQNFTAKMVINKLQWIIPKIIPCLEMQNKLLNIFESNKLI

QMPFISRQFESYNIPNGNREFTWKLGTKYDKIKYIVVAFQCARDNNYLNAGKFDNCGLEG

IYVELNSERYPHECLKFDFDKFNAVQQYNFAKEFRNSYYESTKDYIFMEEDVYYYYYPLL

VFDVSKQNDRIIQSRPDVTIKATFSKSIASSTKCYCLVMSENIVEIKDNRIKVISV

Polinton_Cr1.1

MLEAVDKMLVVDTPIVNDASIQKENLISVFPSSGSSLNQHGEIHFVIETCDQYLHLHKSY

IYMEVDLTKDDGEALAETDLVSMTNNAPMFLFDRAAYSMDGMQVESLMDPGRASLMKGLL

TYSSTMNKQNTFGWILETKDILTYKINKYTKNRKGKITFCIPLTHIFGFAECYNKIVYGR

KHQLSLYRSPENKNSIVSSNVQEFTAKLTINKLQWIIPKIVPSLEMQNKLLNIFESNKLV

QMPFISRQFESYNIPNGNREFTWKLGTKYDKIKYIVVAFQCARDNNYLNASKFDNCGLEE

IYVELNSERYPHDCLKFDFEKFNAVQQYNFAKEFRNSYYESTKDYIFMEEDVYYYYYPLL

VFDVSKQNDRIIQSRPDVTIKASFSKSIAQSTKCYCLVLSENIVEIKDNRVKVVSV

>Polinton_Pd1.2

MASEEKVLKEADLGYEAALDVFQTPVSNVGLQDCKYVQYRATNQFSTEGNVKFRIPAAGA

TYLDLSDIRIRTTLRIVRADGSKVPKQPIRRPEGGGGGGGGGGDGAGDDAGDGDAVEGED

DEREAVPQPGDDGPWSIACINSLSDSLWHQIELRLNDVVVVGGMTGYCYGAMLNTLLEEK

VMTDEELQCAMFYKDTAGYMNTNSLTYGGNEGFRKRAKLMEGSRSVEVQSKLDLDVFKVK

KYLLNGISVDLTLHSTSSAFRLITMNKHDKDYVLEIQDICLIVKQVLPSSQVLIGHQEIL

KSGLSRARYFYMKEDLRKFSVGAGLTSFFAEDAFQSRVPHSLAIAFVAGHSFVGNYNKNP

YYFFHYFVSTINVSLSGCPTARGPLNFDFTRGKYLQGFADLYRGKTKGPNNSQRITLEEF

AAGYSLFVVPLSPQNGENYYPPVRTGSVRIELRFSKPLPESIIMLCRVTYPACLEFDHER

NVYLS

>Polinton_AP

MPESDILDITSQYETDSKITKIEYHSYTPYTTSFNNNDEIRISIQQTDVYPYLHESFIFL

EGTITDATKVKLSNNGYSFLFEQIRLEINGIEVDSTRALGITSSLKGYLSCTPDNYNCYE

NSGWNFKNATQSENEKGEFSACIPLKYWLGLFEDYKRILVNSRLELILTRSHSDLNALSC

KTGATATEGKVVLNKIVWKVPHINVDDEERLKLLKLIEKEKSLFIPFRSFETFEYPELGT

TKKVVWNLKTASKLEKPRFIIIGLQKGRKNLLAKDCSIFDHCNLTNVKVFLNSIAYPYDN

LNLDFSKNNFTLLYNMYTSFQESYYEKSIRNPILSPSTFLANAPIIVIDTSKQNDSATAS

AVDVQLEIEASESLTGVTAYCLLIHDRIVEYVPFTREVRKLV

>Polinton-2_TC

MFDIEKGIEWDEGVVGYKFQSHEAYTARYENTDEIRIPLQEDLCTLPCDSLILIEGQLVK

TDATTNKTVPATETKFVNNGVAFLFSEIRYEVSGVTVDTNTKPGITTTMKNLASLNQSES

LKLTMSGWDLNEEATKPIDGYFQACIPLNRLLGFAEDYKNIMLNIPQELILIRSNSDVNA

LICATNEKARVVIDKIAWLVPHIVPGLKEEVKLTKTIEKDKEIAVPYRSWELHSLPLFPN

TTKCSWPVKISTKFETPRYIIFGLQSNREGQLDKNMSKFDRGDITNMRVFLNNERYPYEN

LNISYKKHHFGILYEMFTNFRSRYYYTDKKETHITPTQFFDSYPLIIIDCSMQKTGLQTQ

SIALRIEFDTDTGISEGTTAYALVISDRAFTYTPLTKSVKQV

>Polinton-4_NVi

MEEVLSIQSPVVFDESVAHYEIHAHQPYTLSNFNNSDEIRIGIQHQDLCVLPSRSSLHIC

GKLQKPDGTAIEGTRFVNNAICHLFEEIRYELNAIEIDRCKNVGLTSLMKGFVSFNPSQS

SAIENAGWLDIAETQRLDDNGYFDVSIPLGMILGFAEDYRKIVVNAKHELILTRSNSDVN

SIVQTQVVVAGANVYEDYQVEITKIEWLMPYVVLSDKHKIKLLNHLEKDRPITMSFRSWE

LYEYPLLPSTSKHVWTVKTANQLEKPRFVILGFQTNRKGRKTANASRFDHCNISNVTLFL

NSQHYPYGNLNLNITNNQYALLYDMYANFQNAYYNKEVEPMLKKADYLSYAPLIVIDCSK

QNESLKQASVDVRLEFEARANIPVGTSAYCLILHDRIVEYNPMSGDVKKIV

>Polinton-4_NV

MATSEVLNFTESPIVDEGIERYEFHEYEPVARTNLNSAGEIRINVEQQDLFTLPSEAYLL

FEGRLTKADGTAYANADAVALTNNGLMHLFSQISYQLSNQDVETVFHPGQATTMLGMLKY

PNDFQLAQGLNQLWYKDSAATAVIADNTGFAVRQAFLIQKPTAKGTFSFIVPLKHIFGFC

DDYNKIVYGFKHTLTLVRKADDDAIFRATAAGAGKVNVDKISLFMPHVIPSDMERMQIYK

TIESKVTLPVAYRARQCDTITVPQSTTFSWRLSVKTSPEKPRYIIVGFQTDKDGSQEANP

AIFDHCDLKNMYIMLNQERYPAVDYNLSFPNQQFSRAYRDAATFSEKFYGMNDLITQSNI

TPSDYKDLFPLMVFDVSKQSERLKSSVVDVQIKATFNAAVPAETEAFAVVVSDRMLQFQS

DGQKMSVVY

>Polinton-2_XT

MAFIHTSSVECAKSELDLFEIPPTQTSVEKSFYVEVQPLSAITDTSPLEFYIAGSGEHYL

DLNNTLLYITCRILKNDNTVPADGARVSLINYPIATLFNQLDVTLGDRLISQSNNLYAYR

AYIETILNYSTDALSTQFTAGLFYKDTPGQHHTRVLDGDNEGFTKRARLMERGKTIELLG

ILHGDIFQQDKLLLSGLDLKIKLTRNKDLFCLMSSEVDPFKVQILNASLFVKRVQVSPAV

RIGHAQGLLTSNAKYIIDRVSMKVFSIPAGSRVCNQENLFLGQLPKLVILGFVDNESFSG

AYNRNPLCFYHNYVCFAALYVDGIQIPSKPYLAEFENGNAIREYMSLVQIAGKKSVDSGF

LIDRESFLGGYTLFGFDLTPDQESSSHFSLIRNGNLRAEIRFSRALDRTVNMIVYGVFDN

IIEVNQRREVLYDFL

>Polinton-2_NVi

MAFLHTHSCECLKSELLLFDIPPTQTTIEGSHWVQYKPISSLTDDSPIEFVIPGNSDEYL

DLAHTMLSLRVSIKSSTSEEDVAEADRAAYRLLTARVGPVNNFMHSLFNQVDVFFNQKPV

SPPTNAYAYRAYIETLLNYGPAAKTSHLSTVIVLVVRYGMEKTTQKTTRGLWKGESY

LLQINLLTLDTSIQMCLIKRSYCSTASKGCVVKSRDNFCLMDPAGSFSVHIEEANLL

VRRVKISPSILLAHAQSLSRATAKYPLTRVEVKAVTMHSGVHGETLDNIILGQLPKRIIL

GFVNNKAFNGDRLLNPFNFEHFNINFLCLYVDGVQVPSKPLQPDFTTRNLYVDAYHTLFS

GTEIHFLNEGNQVTRENYPHGYCLFAFDLTPDLSANDCSHWNLIKHGSVRLEVRFSNALT

ETVNCILYAEYDNILEIDASRQVIIDFSS

>Polinton-2_SM

MICGKSELCIFDRPSPQAVIEYGAFEEVFPMNSITDSRNDVEFYINGSQTEYLDLNDTLL

TVQIKVVNVDRKNLVDTSDVKPNNYMFHTLFKDAILGFNHIKIEGGNNTYAHKALIETIL

NYNGDTKNTCLTPMGYGNSEERKSWIKDSKVFTMCSSLQFDFMDQPKYLLPGVNVHIRLK

RSDSALSLFSKTGEPTCQLLDAKLMIRRVRVESSVLAGHQLGLNSKHAIYPIKTKEIVQF

AIGKGASSFYKEQIFGDRRMPNFILVTFQSESQYNGSYTDSSSKFRHYNVTSLSLSKNTD

YRETYTQDFENNNFCTTYMQSIVRNMGYLDKNLNCGISLDDFKSKYPFFTFVLAPDFDLN

QSQLPQNGNLRLDIKFSKAVEEPVHVIIYGVFENEIQITANRTVLV

>Polinton-2_DR

MALLHTMSEECLKSELDLFTVPLTQTAIEKNAYIEVPPLSAISDTSPLEFFIAGNGEDYL

DLNNTLVFLRLKITNPDGTDIADGAPVGLINYAGATIFSQVDVSLGDRLISQGSNLYPYR

CIIECLINYGKDTLESLFSAGLFYKDTAGHMDTADPAGGNHGLTKRAAFTNASSVLELLT

PLHSDIFFQEKLMLNGVDVRIRMTRGKDEFCLMRSDAVAYKLSILSASLFVKKVAVSPAV

RLGHAQALLSTTAKYPIDRVCVKNFSIPAGSRVCNQENLFLGTLPKSIVIAMVDNDAFTG

TYAKNPFAFKHYDAEFLSIYLDSLQFPSKPLQPDYANGSAVREFYQLVLASGKHLKNHAL

SIDREDFLNGYTLYAFNLTPDEDCGQHISLIKSGNIRLEARFRQPLPRTINLIVYAIFDS

IIEVSNRRQILVDYF

>Polinton-2_NV

MSAARVFDGSQQVANPGVGLFTMPATDVALKDGKFIPYTPSDESVDHIEWYIHPTTTYID

LQKTHIKLKVKITKANGRTLEVNEAAAFVNNVGHSLFRQVKVELNNIPVTLQTDSYPFKA

YISNLLNYTREGQDSFLGCSLWHKDTAGSMDAAVITGVPATNLGLNKRATYTALSAEVGL

IVNLDVDIFKCGRYLLSGVPMKIRLEMSKAAFALMGGGKFVITPGSTLRLYHVTPTDTVA

LTHANELLHHKLAKYPVTTAVVKTEQVENGKLTKKFNDLWNGLIPKRMVFGIITNAAYAG

SYAKNPFNFILTDITNIELRVNGVETPFTSIKNIAGDAIDLYYMMHEGINGKAARTKGLN

INRDEFFGGYGLIVYDLTAAGNASSGYFQPQYKGTVSLDLTFSTAPTEVLTVVLYGEFNN

TMEVDAARSIMYTIQG

>Polinton-2_TC

MFDIEKGIEWDEGVVGYKFQSHEAYTARYENTDEIRIPLQEDLCTLPCDSLILIEGQLVK

TDATTNKTVPATETKFVNNGVAFLFSEIRYEVSGVTVDTNTKPGITTTMKNLASLNQSES

LKLTMSGWDLNEEATKPIDGYFQACIPLNRLLGFAEDYKNIMLNIPQELILIRSNSDVNA

LICATNEKARVVIDKIAWLVPHIVPGLKEEVKLTKTIEKDKEIAVPYRSWELHSLPLFPN

TTKCSWPVKISTKFETPRYIIFGLQSNREGQLDKNMSKFDRGDITNMRVFLNNERYPYEN

LNISYKKHHFGILYEMFTNFRSRYYYTDKKETHITPTQFFDSYPLIIIDCSMQKTGLQTQ

SIALRIEFDTDTGISEGTTAYALVISDRAFTYTPLTKSVKQV

>Polinton-1_TC

MSFLHPCSCECAKSELDLFALPPTQTSIESGQWVHYKTVSSISENSPLEFVVPGGEDYID

LSQTLLSLCIKISKEDGSNYVAEDNIAPVNNILHSMFSQVDVYLNQKLISPPNNTYPYKC

YLETLLNYDSGAKNSHLTCGLWYTDTAGKMNVIGNENIGYAERFKHTSLSKEIDLIGHLN

CDIFNQEKFLINGVEIRLKFARSRDSFALMSSNNLNGKIQITDATLMVRRNRINPSVLLA

HAKALELSTAKYPITRTELKVLTIPQGVQGKSLDNVYLGQLPKRCAVCFVTNKAFNGDYT

MNPFNFENFGLNYLSLYVDGNQIPSKPLQPVFVGGNRKFVSMYHTLFSGTGIHYLNTGNG

ISRDNYADGYSIAVFDLTPDLSSHNGFSWNLIKNGSLRIEVGFTRALTETVNCLVYGEFD

SVLEIDKKRNVIVDYSN

>Polinton-1_XT

MAFVHDSSDECAKSELDIFQIPPTQTSIEKSLYVESQPIAALADNAPLEFFISGSGEYYY

DLNNTLLYILCKIVKQDNTVIGDGARVGFINYPIASLFNQVDITLGDRLISQSDNLYTYR

AYIETLLNYSPQTLSSQFTAGLFYKDTAGHHHDRTPNGENTGFNKRARFTAGSKTVEIIG

PIYGDIFNSPRLILNGLDLKIKLSRNKDAFCLMTADAEHYKVQILQAALYVKRVQVSPAV

RIGHSQALLTTNAKYAIDRVSLKVYSIPAGTRITNHENLFLGQIPKTVILGFVDNDAFSG

SYQRNPLCFHHYNISHAALYVDGQQVPGGRGFQPTFQNDAAIREYMALVHLSGKQKSDNG

ISVDREEFMNGFTLFGFDLSPDQEPGAHFSLVKTGNLRAEIRFAEPTPNTINMIVYSVNA

NIIEINNRREILYDYN

>Polinton-1_SPU

MAFIHCGSEECAKSELDLFQMRPMQTSIENRMYVEVQPLSALSANAPLDFFIAGHEDQYL

DLNNTQLYVCCKIVKADRRNIADDAKVALANYPIAALFNQVDVTLGDRLISQSNGCYPYR

AMIELILNYGEDKLVNQFSAGSFYKDKAGAFEQTDAGNTGFHRRSNLTTQSKVDLLGLL

HCNLFFQEKLLLNSVDVKIKLTRSKDAFCLISGDTNENYKVQILSASLFVKRVKINPGVC

LGHAEALLTSNTKYPTDRVGLKLFSIPAGSKVYNQENLLGGQIPKQIILGFVDTDAQSGN

YTENPFHFKHNHANFVALYVDGERVPAKPLQPDYENGHFVQEYVQLVQATGKNMKYRPFL

FGRREFGGGYTLYAFDLTPDQECADHYSLVRTGNVRAEIRFARALPDTVNMMVYSVFDNV

IEINHTRSMLFDYM

>Polinton-2_DEu

MSNHIECMKSELDLFAPHPTQSSIIRTEEVSYNPIASLDGASSIEFVCLGNGETYRDLSS

VYLRLVVQLRKSDSTGIVGNDVGVVNNILHSIFRSSSVYLNNILVSQSDNNYHYRSYLQT

VLNYGSDASESHLASQGYFPNFGRITAGEYVYPDTNLKLKNLFQNSNKVELFGKIHGDIF

NQPKLLVNNVDLRINFNIEKTAFYLMETGTESNLKILEAQLFMNHITVNPSILLAHHRVL

QTKNAIYPFNKVEVKSFTIYPGNNTLSIDNAVIGQLPNFLAFCMVKNRSYSGNRGLDPFH

FEHFKIQRFNLLVNGVQVPSQALEFDYSNGENVQSSRGYNMLFRSSGIKHYDRGLQITKE

MFDTNSFILAFDLTADHSNSSVCANLITQGTIRIEGRFSEPLTEAVTCLVYCEYDSMIDI

DKHRNIRLLL

>Polinton-1_SM

MISAKSELCLFDRPSPQAVIEHGSFEDIFPMNSITDAGTSSIQFYINGSQTEYLDLNDTL

LYIQLKVVNSLGENITQEADVTANNFFFQTLFKDAELVFNSTKIEGANDSFAHKALIETI

INYNQDTKHTSLGGMGYTEHDLMRKTWIAKSKAFSLCSPLQFDFFDQPKYLLPGVSVQIK

LTRTNPEKSLTCTKFVPKIVLLDAKLMVRRVRVEPSVLAGHQIGLATRNAIYPMRSKEVV

EFSLPIGSSSFYKEQIFGDRRLPNFILVTFQGNKRFSGSFLDTFTMFDHFNVKSITLSKG

SDYREKYSQDFENDNYSTSYMQSIVRNMGYLNKNLNCGISMSDFRNKYPFFTFVLAPDFD

LNQSQLPQSGNLRLDIKFGSPLTESVTVIVYGVFEKEIQITSNRTVLV

>Polinton-1_DY

MSNHIECMKGELDLFAPHPTQSSILRTEEVSYNPIASLDGASSIEFVCLGNGETYRDLSS

VYLRLVVQLKKNDNSVIEGNAVGVVNNILHSIFRSSSVYLNNILVSQSDNNYHYRAYLQT

VLNYGSDASESHLASQGYFPNFGRLTSGKYVYPSSNETLKNIFQNSNKVELFGKIHGDIF

NQTKLLVNNVDLRINFNIEKTAFYLMEKDSESNLKILEAQLFMNHVTVNPSILLAHHHVL

QTKNALYPFSKVEVKSFTIYPGNNTLSIDNAVIGQLPNFLAFCMIKNRSYSGNRGLDPFN

FEHFKMQRFNLMVNGVQVPSQALEFDYSNSENVQSSRGYNMLFRSSGIKHYDRGLQITKE

MFDTNSFILAFDLTADQSNTTICSNLMSQGTIRIEGRFSEPLSEAVTCLVYCEYDSMIDI

DKHRNIRTLF

>Polinton-1_SP

MERIHHMSCECSKSETDLFSIPPTQTTVEKGKWVEFFPLTNISDTTPIQFHLQGSTEEYT

DLSQTLIHLQVKVVNGDGTPLAEDAQVGPTNMFLHSLFSDVDLMLNDRLITPSTNTYAYR

AALETLLTYGPEAKESQLTSALFYKDTPGHMDDGNPLREDAGNMGLKERHRFIKGSKTVD

MVGLLHLDMVFQDKLLLGGVDIKLKLNRSKNSFSLMSSVQNAEYKVIITSASLHVRRVKL

SPETSLLHAKTLETQTAKYPIRRSEVKTFSIPRGNLSFTRESLILGQMPKRLVIGCVSNT

AFNGNYAKNPFNFHHYDLNFLALYADSEQIPWKPIRPNFSAPDPNYILAYQTLFSGINSM

FHDKGNQITRTDYDKGYTLYAFDMTPDLSTGDCFNLRKHGNVRLEMQFARELPETVNVMV

YAEYESVIEIDRNRNVIIDFGG

>Polinton-1_HM

MVKSDIFNITDKLRFDEEIKKYEEYEFTPSVNSNLNSGEIRIFIENSDSLFHPHESYLEI

EGRLVKADGTAYADDYAITLTHNGLMHLFERIEYKFYDSVVESVNFPGIATTMLGMLKYP

NDFQQSKAMNQLWYKDTTATADLVNNTGFXARQQFIIQKPTTKGSFEFSIPLRHIFGFCD

DYDKVFYGLKHELXLLRRSDDNAIFRAAGVAAGKVNITRISLMMRRATPSLVADLELAKI

IKSQETLDIGFRSRFLDKTNVPQNTSFDWRLGLRTTEKPRYILVGFQTNREGNQEQNXSI

FDHCDLXNMWIELNEERYPATNYNLSFPNMKITRAYRHASNFAEDYYNMTNLISLCGITP

SDYRDLYPIMYFDVSKQSERMKDKTVNIKLKAEFNTPVPANTVIYALIISDRIAKITSNG

NRLRFEY

>Polinton-1_NV

MYRNPKYVQRYEDVSFDLETPLVSNVANNAHQTLTGYRIVADNSGEIAPLDWYNARFEVN

FKVQLLADGGNIAVDDHNGIVNGSHSLIKEISVKVNGIPVYDNTQADHTVNIKNLLEYDT

SYAQTTGTNEFYFLDTSRSAEERAGQAGYNKGFAARKALLGTSTTVNTEIPLNRYSFFEA

FEDKLMPNTKVEIQITRNSDATLIWQAGADCRVVITKFKLWVPRIIFNSGGESMYMSKYL

APHKWTYLREMVERSNSGRAQTGSFKITSGINRPRHVFVWISNDANEDSQTVNPFLYNTF

SVANNRTLTSCHLEVGTGNQYPESEYQVSSEMSRVFRDVLGYVNSINDYKGGTLLTRXNF

GSIFPFIYFDLRNQKADIKDGTTKLTFKYRLSAGTNADYSVYALVLYEQDKTVFV

>Polinton-1_LCh

MAFVHCGSEECVKSELDLFTIAPTQTSIEKSLYVEIPPLSALSESAPIEFYIAGNGEDYL

DLNNTLLYIVCKIIKADGSDIARGAKVGLINYPVASIFSQVDITVGDRLISQSNNCYPYR

AYIESILNYSGETLDTQFSTGLFYRDTPGEHEDFDLDGDNEGFKKRNVFTALSRKVELMG

RLHADLFFQEKLMLNGVDVKIKLIRNKDEFCLLSGDADERYKLKIISTSLFVKKKKVKV

SPGVRLGHAEALLTANAKYPIDRVGMKVFSIAAGSRVCNQENLFLGQLPKLIVIGFVDND

SFSGVYHKNPFNFKHYNINFVALYMDGEQVPVKPLQPDFQTGNAIREYCNLIQAAGKHLK

DRPLVINREDFCKGYTLFRFDLTPDQECGDHYSLIKTGNLRAEIRFAEPLPSTVNMIXYA

VFDNVIEINQRRNILFDYM

>Polinton1_SM

MICGKSELCLFDRPSPQAVIEMGAFEDVFPMNSIMDSRTDIEFHINGSQTEYLDLNDTLL

TVQIKVVDKDRKPLGEPSDVIPNNFLFHTLFKDAVLGFNNIKIEGGNSTYIHNALIETII

NYNSDTKNTCLIPIGYGSDDDRKKWIKGSKLFTMGSSLQLDFMDQPKYLLQGVNVHIKLK

RSDSALSLTSASTAPILQLVDAKLLVRRVRVEPSVLAGHQLGLNSKHAIYPLKTKEIVQF

AIAKGSASFYKEQIFGDRRMPNFILVTFQSESQYNGSYLTSSSIFKIFGVKSLTLSKNSD

YRETYTQDFDNDNYCATYMQSIVRNMGYLDKNLNCGITLDDFKNKYLFFTFVLAPDFDLN

QSQLPQNGNLRLDIKFAKATTEPVHVVIYGVFENEVQITANRTVLV

>Polinton-2_SP

MERIHQLSCECSKSEIDLFSIPPTQTTVEKGKWVEFFPLTNISDTAPIQFHLQGSTEEYT

DLSQSLIHLQAKVVNGDGTPLADDAQVGPTNMFLHSLFSDVDLMLNDRLITPSTNTYAYR

AALETLLTYGAEAKESQLSSALFYKDTAGHMDDGNPLRNDDGNMGLKERHRFIKGSNIVD

MVGVLHLDMVFQDRLLLGGVDIKLKLNRSKNSFSLMSSVENANYKVLITSASLHVRRVKL

SPEAALLHAKTLETQTAKYPIRRGEVKTFSIPRGNLSFTRESLILGQLPKRLVIGCVSNT

AFNGDYSKNPFNFHHYDLNFLALYADSEQIPWKPIRPNFSAPDPNYILAYQTLFSGINSM

FHDKGNQITRNDYNKGYTLYAFDMTPDLSTGDCFNLRKHGNVRLELQFASALPETVNVLV

YAEYESVIEIDRNRNVIVDFGG

>Polinton-1_NVi

MSKSHQSSANVKNANTAKDSVSKKPRLKSWWKITCIFFCCQNGRNSHYEIHAHQPYNVSS

YNNSDEIRISVQHQDLCLLPSASSLYVCGRLTKADGTLVKNTKFVNNAVCHMFEEIRYVI

WVSINPSQSLIIHNAGWLDVEEKENLINSEGYFDISIPLSMFICFTEDYRKVVVNVKHEI

ILTRSRNDLNSVIQTPTKVATATTAAEYENFKIELMKVEWLMPYVVLSNQHKIRMLSHIQ

KGKSIDMSFRSWELYEYPLLPTTPKHVWTVKTSNQLEKPRFVILGFQTNRKEAKEINASR

FDHCNISNVKLFLNSQYYPYGNLNLDIERNQYAMLYDMYANFQHAYYDKSIEPMLKKQHF

INYLPLIVIDCSKQNEALKNASVDVRLEFESKDNFPAGTSAYCLIIHDRS

>Polinton-3_TC

MSASKLQVYSSPEIDDSISKEEEHTYSPQVRSFDNNDEIEIIINQRDIWISLFESFIQVD

GEFIPDPVPETGGGNVTLTNNAAAYLFENVSYELNNVELDSVREVGTVSTIKTFLCYGKD

EVRALTLAGWNDQESQQLKTFNETDNTFSFRIPLSYLLNLPFDYHRIVSGHQKLRLIRSR

NDANCFISTGTRKATLKINNIELKAKHVYPNDEIKLTILEGINKDRVISLPFRKWEIHEL

PSVRQTNNDIWRVKTSTQLERPRFIIVCFQTNRKNNPKSDVTLFDHCDVRSVRLWLNSNV

YPYETWKLQFAKNKYLEAYQAYVDFYKEFNGKDRAEPILSYTDYTKRPIFVLDCSKQNEA

IRSSTIDISLEFESDNNFPADTRAYCIIIHDRIMQYYPLTGIIRQLI

>Polinton-3_SP

MSLLHDKSDVCCKSELDLFTLPFTQMSILKGPWVEYHPVSNITDSAPIEFNVAGTAEEYV

DLSQTMLAVTAKITNPDNTNLAQEAPVGPVNLFLPSLFSQVDVMLNEKLVSQPSNTYPYR

AMLESIMHYGKETKDSQLTQQLYYKDAAGKMDLLNPLLAGENVNEGLKKRHEFIVDSKVL

SMLGPIYGDLFFQERLLLPGVDLKLKLNRSKDAFCLLSSNAEGNYKVKILSAALYVRRVK

TNPSVMLNHAKMLEKANAKYPLNKVDVRSFTIPAGNMSFNKDNLFLGHLPNRIVVGFVDN

DAYNGTYGKNPYNFKNLLLNYVGVTVDGESIPMKPLRPNFREGPGQDFIHAYNSLFMGSN

RLFQDKGIDINREEYARGYTLFAFDLTPDLSDGCHLNLVKQGNLRLELQFDNPLPNTVNC

LVYSESQGLIQIDRSRNVVYDYQG

>Polinton-1_DBi

MSTHIECMKSELDLFAAHPTQSSILRTEEVSYNPIASLDGASSIEFVCLGNGETYRDLSS

VYLRLVVQLKKNDNNSIEGNAVGVVNNILHSIFRSSSVYLNNILVSQSDNNYHYRAYLQT

ILNYGSDASESHLASQGYFPNFGRITAGKYVYPDSNETLKNMFQNSNKVELFGKVHGDIF

NQTKLLVNNVDLRINFNIEKTAFYLMETGTESNLKILEAQLFMNHITVNPSILLAHHHVL

QTKNALYPFSKVEVKSFTIYPGNNTLSIDNAVIGQIPNFLAFCMVKNRSYSGNRGLDPFQ

FEHFKIQRFNLLVNGVQVPSQALEFDYSNAEKVQSSRGYNMLFRSCGIKHYDRGLQITKE

MFDSNSFILAFDLTADQSNSTICSNLISQGTIRIEGRFSEPLAEAVTCLVYCEYDSMIDI

DKHRNIRTLF

>Polinton-1_CI

MFIHKNSCECLKSELDLFSAPMTQTSVEDGMWVANGPQNGLSDSGTLEFLISGTKEHYID

LANSYMHIQVKIVNADGTNLAEDAEIAPVNNFFHSLWSQIMLSLNNREVTSSGSMYPYRS

YIESLLTFGSAAKSTYLTGALFYKDTPGHMDSIAAANVGATTRRAFAAESKVIDMQSKLH

LDMMFQQRYLINNVDVKLKLTRSRDAFATIGVAGFKIKILAAVLHVRKVKISPSVQLGHI

TALSKGLCLYPILRAELKTLTIPRFNQSISHDNLFLGQLPRRVILGFVDNDAFNGRIDKN

PFHFKTYGLSSLSLHSNGKQVPAKELTPNYGVGTYIRSYMSLFTGLNTFYTNASNGISRE

EYPRGFTLYAFDLTPDLASSGHFDLLKNGDLSLEAKFSEALPTSVNVIIYAEFQNLIQID

RQRSVLTDFSA

>Polinton-1_CPB

MAFVHCGSEECTKSELDLFQIAPTQTSIEKSIYIEVPPLSAITESAPIDFFIAGNGIDYM

DLNNTLLYLCCKIVKGDGTELAVDAEVGLVNYPVASIFSQLDVTLGDRLISQSNNCYPYR

AFIESVLNYSDDTLATQFSAGLFYKDTAGQHEETELDGGNLGFVRRAKLTAESRTVELLG

HLHSDLFFQEKLLLNGVDVKIKLTRSKDAFCLMGSAAEGFKLRIVSASLFVKKVRVAPGV

RLGHAEALLTANAKYPVDRVGMKVFSIPAGSRVSNQENLFLGQLPKTLVLGFVDNDAFSG

SYAKNPFHFKHYDINFVALYVDGEQIPTKPLQPDFEAGRCVREYMNLVQTAGKHMKDRSL

LIDREEFAQGYTLFAFDLSPDQECADHYSLIKTGNLRAEIRFGKALTVTVNMIVYGVFDN

VIEINQRRNVLFDYM

>Polinton-1_DAn

MSNHIECMKSELDLFAPHPTQSCILKTEEVSYNPIASLDSASSIEFVCLGNGETYRDLSS

VYLKLVVQLKKIDNSNIEGNAVGVANNILHSIFRSSSVYLNNTLVSQSDNNYHYRSYLQT

ILNYGCDASESHLATQGYFPNYGTQTPFKSDGCLILKNIFQNSNKVELFGKVHGDIFNQP

KLLVNNVDLRITFNIEKTAFYLLEDGTESNLKILEAQLFMNHVVVNPSILLAHHHVLQTK

NAIYPYNKVEVKSFTIYPGNNTLSIDNAVIGQIPNFLVFCMVKNRAYSGNRGLDPFNFEN

FKIQRFNLLVNGVQVPSQALEFDFSNSNNVQSSRAYNLLFRACGIKHYDRGLQISKEMFD

KNSFILAFDLTADHSNSSVCSNLISQGTIRIEGRFSEPLDEAVTCLVYCEYDSMIDIDKH

RNIRVLL

>Polinton-1_CPB

MAFVHCGSEECTKSELDLFQIAPTQTSIEKSIYIEVPPLSAITESAPIDFFIAGNGIDYM

DLNNTLLYLCCKIVKGDGTELAVDAEVGLVNYPVASIFSQLDVTLGDRLISQSNNCYPYR

AFIESVLNYSDDTLATQFSAGLFYKDTAGQHEETELDGGNLGFVRRAKLTAESRTVELLG

HLHSDLFFQEKLLLNGVDVKIKLTRSKDAFCLMGSAAEGFKLRIVSASLFVKKVRVAPGV

RLGHAEALLTANAKYPVDRVGMKVFSIPAGSRVSNQENLFLGQLPKTLVLGFVDNDAFSG

SYAKNPFHFKHYDINFVALYVDGEQIPTKPLQPDFEAGRCVREYMNLVQTAGKHMKDRSL

LIDREEFAQGYTLFAFDLSPDQECADHYSLIKTGNLRAEIRFGKALTVTVNMIVYGVFDN

VIEINQRRNVLFDYM

>Polinton-1_CPB

MAFVHCGSEECTKSELDLFQIAPTQTSIEKSIYIEVPPLSAITESAPIDFFIAGNGIDYM

DLNNTLLYLCCKIVKGDGTELAVDAEVGLVNYPVASIFSQLDVTLGDRLISQSNNCYPYR

AFIESVLNYSDDTLATQFSAGLFYKDTAGQHEETELDGGNLGFVRRAKLTAESRTVELLG

HLHSDLFFQEKLLLNGVDVKIKLTRSKDAFCLMGSAAEGFKLRIVSASLFVKKVRVAPGV

RLGHAEALLTANAKYPVDRVGMKVFSIPAGSRVSNQENLFLGQLPKTLVLGFVDNDAFSG

SYAKNPFHFKHYDINFVALYVDGEQIPTKPLQPDFEAGRCVREYMNLVQTAGKHMKDRSL

LIDREEFAQGYTLFAFDLSPDQECADHYSLIKTGNLRAEIRFGKALTVTVNMIVYGVFDN

VIEINQRRNVLFDYM

>Polinton-1_DGr

MTNLLTVREKPYSDESIIKKEYHSYSPYLQSFKSNDEVRITIQNQDLYVLPSESFINIEG

LISLANNAPTGGGKLKNNCMSYLFDEIRYELNGIEIDRTRFVGTSTTLKNYISLNKPESR

MLENSGWFNGNEKLVPNDGYFNFSIPIKYLLGFCEDYNKVLLNCKHELILLITKNMDDVY

HPKIGSIEYKLNITNITWKVPHITLSDEKKLLMLNITKNGGSLPLAFRSWDCYLNPTLAE

GTHHVWNVKLAANRERPRFAIIAFILDNKLIQNQLRNLKVYLNSETYPYDDLNINFSRER

FATLYDMYANFQASYYMKQSEPLLTPKEFKEKAPIVVIDLTHQNESVKTGPIDIRVSIEL

ESASVRNTKVYCLLMHDRLVEYSPLTGLVQRIV

>Polinton-1_CPB

MAFVHCGSEECTKSELDLFQIAPTQTSIEKSIYIEVPPLSAITESAPIDFFIAGNGIDYM

DLNNTLLYLCCKIVKGDGTELAVDAEVGLVNYPVASIFSQLDVTLGDRLISQSNNCYPYR

AFIESVLNYSDDTLATQFSAGLFYKDTAGQHEETELDGGNLGFVRRAKLTAESRTVELLG

HLHSDLFFQEKLLLNGVDVKIKLTRSKDAFCLMGSAAEGFKLRIVSASLFVKKVRVAPGV

RLGHAEALLTANAKYPVDRVGMKVFSIPAGSRVSNQENLFLGQLPKTLVLGFVDNDAFSG

SYAKNPFHFKHYDINFVALYVDGEQIPTKPLQPDFEAGRCVREYMNLVQTAGKHMKDRSL

LIDREEFAQGYTLFAFDLSPDQECADHYSLIKTGNLRAEIRFGKALTVTVNMIVYGVFDN

VIEINQRRNVLFDYM

>Polinton-1_DPe

MTTHIECMKSELDLFEPHPTQSSILRTEEVSYNPIASLDSASSIEFVCLGNGETYRDLSS

VYLRLVVQLRKSDNTNIEGNDVGVVNNILHSMFRSSSVYLNNILVSQSDNNYHYRSYLQT

ILNYGSDASESHLATQGYYPNYGKLLSGRYVNTDGSIMLKNIFQNSNKVELFGKVHGDIF

NQPKLLINNVDLRITFNIEKTAFYLMESETESNLKILEAQLFMNHITINPSILLAHHHVL

QTKNAIYPYSKVQVKSFTIYPGNNTLSIDNAVIGQLPNFLAFCMVKNKSYSGNRALNPFN

FDHFKIQRFNLLVNGVQVPSQALEFDFSNSENAQSSRGYNMLFRSSGIKHYDRGLQITKE

MFDKNSFILAFDLTADHHNSTVCSNLISQGAIRIEGRFSEPLTEAVTCLVYCEYDSMIDI

DKHRNIRVLL

>Polinton-1_DR

MALLHRMSGECIKSELDLFTVPLTQTAIEKNTYVEVPPLSAISDSSPLEFFIAGSGEEYI

DLNNTLLHLRLKITKPNGGEIADPAKVALINYPAATIFSQVDVSLGDRLISQSSSTHPYR

CIIESLINYDKDTLESTFSAGLFFKDTAGHMDVKDPLGNNQGLLKRSTYTSRSKIVDLMG

PIHSDIFFQEKLMLNGVDIKIRMIRGKDEFCLMRSDDVAYKLKIVSASLFVKKVSVAPNV

RLAHAQALLSTNAKYPIDRVCLKNFSIPEGARVSNQENLFLGTLPKSIVLGMTDNDAFTG

SYDKNPFAFKHYDLEFLAIYVDGQQIPAKPLQPNFTDGSVVREFYQLVTATGRHLKNHAL

SISRSEFARGYSLYAFNLTPDEDCGQHVSLIKSGNIRLEARFRQPLPNTINLIIYSVFDS

IIEVSNRRQILVDYY

>Polinton-3_NV

MAQAISEKINPNRIPREPFGLKAESSLNRITFNPSSASPGETLYINIPKLAENVVIVPGS

VSLLFDLNVTGHANNTLVNNVGRNLVSRLKILFGGETLQDTQRYDLFQTYHDLYLQAEDR

EDRIKQGISSENMRKLRTNAGDKATSDAKEVALAAVHNTKYCIPLDHPILGEHGVFYPKA

LPHPLIFEITLAPVSDVVVYADTVKTPTYTITNLELEYACISSEYLAREALSAYQVGRGF

FYENVILHKTFTISKPNDGVINEHINLPRRSMTGILCLFTESYTGGARDSEKFVNPSITS

ININVDGMPNRLYSKGMTPPDLWESVKKRFGREGVKQKDFYANNKFALWVDLRAHPDNSI

HGGGLVLNNTRDGVKLEMKRKVGGTGNITCYMFVVADALMEVMNSNLRAIMY

>Polinton-2A_NV

MSDATVFSGSQQVANPSVGLFTMPATDVALKDGHFIPYTPTDEGSEHIEWYIHPPSTYVD

LKKTHIKMKVKITKADGANLTAGEHAAFVNNAGHTMFRQIRVELNNRVVTLQTDSYPYKA

YISNLLNYTREGQESFLVCGIWHKDTAGHMDIAEVSGAGATNEGLKKLASYTAESAEVGI

IIPLDVDIFKSGRYLLSGIPMKIRMEMSKPNFALMGGGKFVISPGSTLRLYHVTPLETIA

LGHANELLHNNLAKYPVTTAVVKTEQVESGKHTIRFNDLWNGLIPKRMVFGIVTNEAYAG

SYTKNPFNFHFPNVTNIEVRVNGNETPISNIKNIAGDAIDLYYMMHEGVNGQAARAKGLN

ISREEYFGGYGLVVYDFTPAGNSSSGYFQPQYKGTVSLDLNFSTAPTVVLTVVLYGEFNN

TMEIDNGRNVMYDIQG

**Minor capsid proteins (PX)**

>Tlr_penton

MNYIFIDSSKINPSCFYNFQVQLNQKVRVTKYIKLIKAVIPYDDYLINQYNNTSQINSKT

YSIPIGIYDVPSLINQIKSLVTPDLSSFLVQFSSLTYRITFSASTNFTMSFNQSLANILG

FQQTQFQSNSSYTTTKTPSINSPQTILINIKDIPDSQIIQAQNYDVDFSFMILNISNRGQ

NIQYENRSDENTIEITGDIRQISVQLYKNDGQFFMINSGIQLSFQYE

>Polinton_AP

MHESITLSFTGNSTVLSANYFPSLNVYGDSEIALLSLQTFNSFPNIINPTNNRLKIETIP

PKRKKDDYHVFEFCLEDGCYEIEDINNYMAKELSQVNNDHGTHLTFSIRMDPVDFRTYIK

CNGILRFNTSFSIAPVFGYRKKDCGPFHEEHRSDKATNLNTINSIKVMCNIAHGSFNNQL

QSHSIYEFFPSGRTGTKVVQSPVNLIYYRLNKTDINSITVQLVDQNNNPIDNFNETLTVV

LHIKRHGSDH

>Polinton-1_NV

MENIVERAVYISSRDREARGVSRPDDFTIKMNPSLRLSNDARHEIAVDKISMTYSWHNIN

PEYGNDSVKYSPDGGATWTTVTFPSGMYSYHDLNDFLHQDMKSNGHVGVADGKDKFYIDI

VFVLTTYKVVVIVGDKYELDIRGTKFGELIGFDPKVISKTEYGSKLPNITNSIDTLHINT

DLITDSIVGGRASNTLFVIPTDNLSRSYPFSVQPTRALFNNVAANLISSMRFYVVDSIGR

PINLNGIEWHMTLILRSIY

>Polinton-4_NV

MEQLLEKIARNTEPKKSFYILVSDKSTRIRTKFNPLLQLDKSKQYEMALVNLETYYSFPN

IDSTNNNFRYSPDNGATWFNIDIPEGSYEITDINDYVQRIMKDNGHYDSANDEYYITIEP

NNNTLKSVVDISANYKVDSTTANSIRTVLGFNSQVYSEGYNESENIVNILSVNSLRVTSD

VIASSYTNGGTENIIYSFFPSVGPGYKIVQEPLNLIYLPITLGTISQMETKLVDQNGKLI

NLRGEELSIRLHIREA

>Polinton-4_NVi

MEDSLTLSLSGTSAVLEAQYFPPLELSANKSYVLGLVELLTFNSIPNIDTGNNKFYVGGE

VIILPTGSYEIEDIEKSLKEALTPKGITLKLKPNNNTLRCMIKCNRSIDFQPDDSIGKLL

GFTSRVLSPNTDYESDLPVTILKINALRVECNITSGAYINEHKVHTIHEFFPAVSPGYKI

IEVPSPIIYLPVTVKTINNLQLHIVDQDGHLVNFRGEVITIRLHIKSV

>Polinton-3_NVi

MIESLTLSLSGTSSVLETQYFPPIELDSDKMYVLGLVELLTFNSIPNVDFKSNKFYVGQE

IIELPTGSYEIEDIERTLQEILSLKNITLIIKPNNNTLRSVITCSHEVDFRPKDSIARLL

GFTPRLLKPYITHNSDLPVAILKVNALRVECNITSGAYINQNQVHTIHEFFPAVPPGYKI

IEVPKQIIYLPITVKIIDHLQLRIVDQDGDLVNLRGEVITIRLHLKNV

>Polinton-6_NVi

MENSLTLTLSGSSSILEAQYFPPIELSPHKQYVLGLVELLTFNSIPNIDKGNNKFYVGKE

EIVLPTGSYKIQDIDSXLREILTKKKISISIQPNNNTLRSIIKCNRKIDFRPQDSIGALL

GFTQRVLQENXKHSSDLPVAILKVNALRIECNITSGAYINGQLAHTIHEFFPAVPPGYKI

IEVPSQVIYLPITVKSIDHLQIRIVDQDGHLVNFRNETITIRLHLKPT

>Polinton-2_TC

MQIISLVESKMLFVLTGKSAILSADFNPPIDVSDGVYELGVTNFEVYNSIPNIDEENNKF

FFGDVEFKIPTGCYQLTDINNYLQHVIEKQFSNDLLSITANNNTLHTHIKATKDVDFTKP

NTIGPVLGFNSQIVPKNIGKDSDNIADIMKLNSIMIECNITIGSFKNGEPVHIIYQFFPN

VPPGFKIVQSPDHVIYLPISVKTIRNITLKIIDQDEKLVNFQQETVTVGLHLQKKEENGY

>Polinton-1_CI

MVEDFYVTLPSNASLEYFPNNTLTNYVTRLPKEFHLQGTWVVGLTEVAFPYSWYTIPQQA

VTMKIRRGPDLGVIEADISPGYYNSPKELIESIVKLFTDVGYANNIEVSYNDLTGKVRVN

VHKKGWYLEFSDVLSRMLGYDKKRLRRGSHEAERVCDISLDMSLLYFYSDIVQGRSVGDT

LAPLLAIVPVKGRPREAVYQRFDYPCYHPIQNKVFHTIEVDIRDSLGKKILFQRGRVCVT

LHFKRVNKA

>Polinton-5_SP

MASHFYVSLPSNSSMLYYPDNKTCKYTTKLHAPLSLHDDYEVGLSEIQFQCTWYNVRKGN

NTLYIYDRETVTDSVPLLRQITVQEGYYMNGVDFIHAINAALDVATREKVNFSYIEASRK

CAIQVQPGIGIVLPCSLARMLGFFEKCNLTQSTESPSPVDVHMYFHSFYVYSDILQFRHV

GDTSVPLLRTIAVMPRSRQENIVNTYIAPHYLPLKLFNFETIDIIITNETGEVVPFERGK

VLVTLHFRERSSRLS

>Polinton-2_CI

MTQDFYITLPSNASLNYFPNNTLTNYVTRLSKSINLEGTWVVGLSEVTFPFSWYNVSKKA

SRGLKVKQRVGGITGIDISPGYYESPRLLINNIASTLRGVNFTDYIQISYIPITGKASVA

VAKGWYFEVTEPLKQMLGFRKKRLGYGYHESDKVCDVNLDLSTLYIYSDIVEGRAVGDTL

APLLGIVSVKGKYGKAIYQRFDKPSYHPIQNKNFHTIEIDLRDSTGDPILFERGRVCVTL

HFKRVNKI

>Polinton-1_SP

MESEDQVYLTLPSNSSFDYFPNNTLTSFTTKLATPLVLRGEYEVALVDIIYPHSWTNVNF

TNNKYSFSIGDQVITVGRVPVGHYRDAQSVCTAFNESLPANLRNKASFNINPSTLKVRAE

IQPDSGVYLSEGLGQLFGFPEGTLCTNQEGKFLPDLNGGLFAMYVYTDIIENQRVGDISA

PLLRIVAVDHKRAGEVVDRSYQTPHYLPVKSKYIDTIHIDIRSDFGDKVPFQNGKVVLKL

HFRSVRRRSFHL

>Polinton-3_SP

MGSHFYMTLPSNSSMQYYPNNKTCKFTTKLHTPLSLNGDYEVALVEIQFQCTWYNVREIN

NTITVFNKNTEDGVPLVRNVTLPTGYYYTGEELITMINASLDQDWRQRVGLFYKKSSRKV

YVQLRADTGLLIPCNLARMLGFDRECHLLESTESPMPVDPHVEFHTFYMYSDIVQYQHVG

EVSVPLLRTIATKARKDQYNSVSTYNTPHYVPLKLYNFETIDIILTTESGEVVPFERGKL

IVKLHFRERSPSL

>Polinton-2_SP

MESEDQVYVTLPSNSSFDYFPNNTLTSFTTKLATPLVLRGEYEVALVDVIYPHSWSNVNF

TNNKYSLSITDQVITVGRIPEGHYREALSICTAFNQSLPPHLRNKACFTINPSTLKVRAT

IQPDTGIYLSEGLGQLLGFPEGTLRANQEAKFLPDINGGLFAMYVYTDIIENQRVGDTSA

PLLRIVAVDHQRAGEVVERTYQSPHYIPVKRKYIDSIHIDIRSDFGDKVPFQNGKVVLKL

HFRSLRRPTFGFNKI

>Polinton-1_XT

MDEGSFYMTLPSNASSKIYPDTTKLAKSVDLRGPWEVALTEIQYPHTWNTFDPHEGNFVV

GKQDDLLKEYHIKSGYYNTINEVVKAINARLDSLKIPHEHIKLRYDDLERSVSVSESPIY

TFAPGEKLAHILGMDGYIAPYGTSLPKVKKIYADIKAGFYTMFVYSDIIQHQLVGDSYVQ

LLRTVEISGKNNEIITQRYTRPDYIPVCKQHFDSVAISIYSDQCKPVKFKYGKCLVRLHF

RPRKELSY

>Polinton-2_XT

MEEEAFYITLPSNASLSTFPQNEISNYTVKLSKPVMLRGEWEVGLTEIQYPHTWNTFETD

EGLFYVGIHGGPLKELNVKPGLYNSVKDLVKAINDKIEAYKSPTYDVKLRYDELERIVTV

KGTHSFLAGNKLTHILGIDSNNFNDSINGQLCADIKAGFYTLFVYTDIIRPQRIGEFYTP

LLRTVPITGSNNEIVTQQFIKPDYLPVSKHHFDNITIEIKSDQNRNVSFKYGKAIVKLHF

RPRRAYY

>Polinton-1_CPB

MSDGGFYITLPSNASSAVFPQNTISNFTIRLIKPLDLPGAWEVGLAEIQYPHSWNTINED

TPFEITFGATTWNFILRRGYYSTIPELLEHMNSNVARHPGPPEVVMNYDPVGRKVRLKST

DFMYVFSTDGELANILGLGHKRNVQKFPFSADITGGFNSLYLYTDIVEHQFVGDFSVPLL

RCVPVRGRNNEFVTITYDKPHYVPVSKHHIDTITIEIKTDQNRHVSFRFGKVIVKLHLRP

RRERGF

>Polinton-N1B_NV

MVVLHTVLTKGEQTLFFERPIRRPRFVALRQCSLFNSWYNLEREHQITTIAALPPIATLP

AGHHTAESIVETINRAESKILTAELDPLTGKIALMSNGVAFLNAELCALFGLEAKDDQLG

ERGLALPLGEPALKVALPKIEALYICCDIVDRTQCLSLGEPSNVLACPETRGRPHEKVVY

GPDVPVCVAASSSEFVSSIQIWIRDDCGRRVDFKGKPVRLVLELTFRHNKHGKQRR

>Polinton-N1A_NV

MFNTMVVLHAVFTGGELTLFFEQPIRRPRFVALRQCSLFNSWYNLEREHQITTIASLPPI

VTLPAGHHTAESTVETINHVKSQILTAKLDPLTGKIVLVSTGIVFLNAELCALFGLEAKD

DQMGERGLALPLGDPPLKVTLPKIEAIYIFCDIVDRSQCLAFDEPSNVLACLETRGRPHE

KVVYGPDIPICVAASSSEFVSSIRIWIRDDRGRRVDFKDKPVRLVLELT
